# CT-based radiomics in predicting pathological response in non-small cell lung cancer patients receiving neoadjuvant immunotherapy

**DOI:** 10.3389/fonc.2022.937277

**Published:** 2022-10-04

**Authors:** Qian Lin, Hai Jun Wu, Qi Shi Song, Yu Kai Tang

**Affiliations:** Department of Oncology, Xiangya Hospital, Central South University, Changsha, China

**Keywords:** radiomics, pathological response, NSCLC, biomarkers, lung cancer, immunotherapy, neoadjuvant therapy

## Abstract

**Objectives:**

In radiomics, high-throughput algorithms extract objective quantitative features from medical images. In this study, we evaluated CT-based radiomics features, clinical features, in-depth learning features, and a combination of features for predicting a good pathological response (GPR) in non-small cell lung cancer (NSCLC) patients receiving immunotherapy-based neoadjuvant therapy (NAT).

**Materials and methods:**

We reviewed 62 patients with NSCLC who received surgery after immunotherapy-based NAT and collected clinicopathological data and CT images before and after immunotherapy-based NAT. A series of image preprocessing was carried out on CT scanning images: tumor segmentation, conventional radiomics feature extraction, deep learning feature extraction, and normalization. Spearman correlation coefficient, principal component analysis (PCA), and least absolute shrinkage and selection operator (LASSO) were used to screen features. The pretreatment traditional radiomics combined with clinical characteristics (before_rad_cil) model and pretreatment deep learning characteristics (before_dl) model were constructed according to the data collected before treatment. The data collected after NAT created the after_rad_cil model and after_dl model. The entire model was jointly constructed by all clinical features, conventional radiomics features, and deep learning features before and after neoadjuvant treatment. Finally, according to the data obtained before and after treatment, the before_nomogram and after_nomogram were constructed.

**Results:**

In the before_rad_cil model, four traditional radiomics features (“original_shape_flatness,” “wavelet hhl_firer_skewness,” “wavelet hlh_firer_skewness,” and “wavelet lll_glcm_correlation”) and two clinical features (“gender” and “N stage”) were screened out to predict a GPR. The average prediction accuracy (ACC) after modeling with k-nearest neighbor (KNN) was 0.707. In the after_rad_cil model, nine features predictive of GPR were obtained after feature screening, among which seven were traditional radiomics features: “exponential_firer_skewness,” “exponential_glrlm_runentropy,” “log- sigma-5-0-mm-3d_firer_kurtosis,” “logarithm_skewness,” “original_shape_elongation,” “original_shape_brilliance,” and “wavelet llh_glcm_clustershade”; two were clinical features: “after_CRP” and “after lymphocyte percentage.” The ACC after modeling with support vector machine (SVM) was 0.682. The before_dl model and after_dl model were modeled by SVM, and the ACC was 0.629 and 0.603, respectively. After feature screening, the entire model was constructed by multilayer perceptron (MLP), and the ACC of the GPR was the highest, 0.805. The calibration curve showed that the predictions of the GPR by the before_nomogram and after_nomogram were in consensus with the actual GPR.

**Conclusion:**

CT-based radiomics has a good predictive ability for a GPR in NSCLC patients receiving immunotherapy-based NAT. Among the radiomics features combined with the clinicopathological information model, deep learning feature model, and the entire model, the entire model had the highest prediction accuracy.

## Introduction

Lung cancer is a heterogeneous malignant disease arising from the bronchial epithelium or alveolar tissue, usually caused by smoking, varying environmental exposures, and underlying genetic susceptibility (1). According to the 2020 global burden of cancer statistics provided by the International Agency for Research on Cancer (IARC), lung cancer ranks second in global incidence and first in mortality. In China, lung cancer is the most common cancer with the highest incidence and mortality rate. The most common subtype is non-small cell lung cancer (NSCLC) with an incidence rate of about 85%, while the 5-year survival rate is only 10%–20% (2, 3).

Currently, surgical treatment remains the mainstay of treatment for early-stage and locally advanced (stages I and II and some with stages IIIA and IIIB) NSCLC(NCCN) (4, 5). However, patients experience high rates of local and distant recurrence postoperatively, suggesting that systemic therapy is necessary to improve cure rates. Neoadjuvant therapy (NAT) is a form of cancer treatment and refers to systemic therapy given before surgery, including neoadjuvant chemotherapy, chemoradiotherapy, targeted therapy, and immunotherapy. However, the technical definition of NAT usually refers exclusively to neoadjuvant chemotherapy and is distinguished from adjuvant chemotherapy after surgery. NAT (6) reduces the rate of distant disease recurrence by taking advantage of the damaged lymphatics and vasculature resulting from surgery, thereby increasing local drug concentration. Effective antitumor therapy can shrink the primary lesion and downstage the tumor stage, reducing the need for extensive surgery leading to organ preservation and improved quality of life. Moreover, patients are generally in a better situation and less likely to experience acute toxicity before surgery, and currently, receiving systemic therapy is more well-tolerated. By observing the radiological and pathological responses following NAT, tumor sensitivity to chemotherapeutic drugs can be understood, which provides a reference for the choice of the postoperative treatment regimen. Effective NAT can minimize the proliferative capacity of tumors at the time of surgery and reduce the risk of intraoperative dissemination of cancer cells, and even a small proportion of patients can experience major pathological response (MPR) and complete pathological response (CPR) (7).

Lately, with the wide use of immune checkpoint inhibitors (ICIs) such as anti-programmed death-ligand-1 (PD-L1) and anti-programmed death-1 (PD-1) antibodies in advanced NSCLC, patients have had significantly improved quality of life and good prognosis, making immunotherapy (8–10) a new option for the treatment of resectable and potentially resectable NSCLC. In previous clinical studies, either single-agent ICI or immune doublet combination NAT significantly led to higher MPR and CPR rates and lower complication rates than neoadjuvant chemotherapy (11, 12).

In an exciting phase II study of ICI with chemotherapy, the MPR and CPR rates were 83% and 63%, respectively, with 90% of patients who underwent resection achieving clinical stage (13). Although the large-scale phase III study is still ongoing from the results of the abovementioned phase II studies, patients treated with neoadjuvant ICI and then surgery had a similar adverse event as compared to chemotherapy combination but with better pathological remission (residual tumor cells in tumor bed ≤10%) and improved quality of life (14).

In general, the higher the MPR/CPR rate, the better efficacy of NAT (15, 16). If the pathological response information can be evaluated before surgical resection, it will guide the type of surgery. Therefore, developing noninvasive assessment response models can help identify patients with good responses who may benefit from local excision. Those who achieve CPR may benefit from the watch-and-wait or nonsurgical strategies.

Imaging modalities such as computed tomography (CT), magnetic resonance imaging (MRI), and positron emission tomography (PET) have become routine in the clinical management of patients with tumors such as lung cancer. Lesions detected through these imaging modalities are described and analyzed only based on simple qualitative (e.g., shape, location, spiculated lesion, and lobulation) and quantitative (e.g., size, volume, density, signal, and standardized uptake values) features. The radiological diagnostic accuracy is closely related to the radiologists’ experience, with marked subjective differences. In 2012, Dutch investigators (17) first proposed the concept of radiomics, hypothesizing that the cellular and molecular heterogeneity of tumor cells can be reflected by quantitative imaging microheterogeneity. They also concluded that when extracted with radiomics, these features can transform image data of interest regions from medical imaging into quantitative data through high-throughput algorithms. The general procedure of radiomics is as follows: 1) Acquisition of medical imaging data; 2) Region of interest (ROI) segmentation and feature extraction; 3) Feature selection, model building, and validation; 4) Statistical data analysis. Since then, the concept of radiomics has been widely studied in the differentiation of benign and malignant lesions (18), in the preoperative prediction of lymph node metastasis in lung cancer (19), and in the assessment of the mutational status of genes such as Epidermal Growth Factor Receptor (EGFR) (20) and anaplastic lymphoma kinase (ALK) (21). More radiomics studies include the prediction of treatment effects and prognosis in cancer (22, 23) and even in non-neoplastic diseases such as the early diagnosis of Alzheimer’s disease (24) and the rapid radiological diagnosis of coronavirus disease 2019 (COVID-19) pneumonia (25, 26).

With the development of computer software, computational power has significantly improved, and in recent years, artificial intelligence (AI) technology based on deep learning (DL) algorithms has been vigorously developed and has gradually begun to be applied in medical research. Currently, it is mainly based on medical images using computer vision technology to solve clinical tasks such as lesion segmentation and disease classification (27–29). In the processing of medical images, the most widely used DL network is the convolutional neural network (CNN). A CNN is a computational method for learning relevant features from image signal intensities proposed based on the working principle of the human nervous system. The ability to directly utilize high-dimensional numerical information in images from a large enough number of training data and identify image features with a high degree of representativeness creates and selects a large amount of abstract information at the hidden layer, which is defined as DL. DL features can be used more comprehensively to accomplish segmentation, classification, and other targeted tasks (30).

Building entirely new DL models requires large amounts of annotated data to be used as training data; however, the sample size of most radiological data is often limited. The method of using a trained DL model on other data sets for a target data set and for extracting the features of the target data set is called transfer learning. Transfer learning offers the possibility of DL for small samples of medical data. Many studies have confirmed that this is an effective and superior way to conventional machine learning (27, 31).

This study aims to extract radiomics features and DL features from CT images of patients with NSCLC before NAT with ICIs and after NAT with chemotherapy, then combine the features with clinicopathological information of patients.

Combining clinicopathological feature signature and DL feature signature that could predict pathological remission after NAT with ICIs in NSCLC patients was done through feature screening. Also, binary logistic regression analysis constructed a prediction model integrating traditional radiomics feature labels, DL feature labels, and clinicopathological information.

Finally, a nomogram was constructed to visualize the model, achieving a precise assessment of pathological remission after NAT with ICIs in NSCLC, thereby providing an adjuvant tool for developing individualized treatment regimens for patients.

## Materials and methods

### Study participants

Clinicopathological information was retrospectively collected from 83 patients with pathologically confirmed NSCLC and treated with immunotherapy-based NAT between 1 March 2020 and 1 January 2022.

Inclusion criteria include patients with pathologically diagnosed NSCLC through either image-guided biopsy or bronchoscopy-directed biopsy; potentially operable stage Ib–III NSCLC as per the 2017 Union for International Cancer Control (UICC)/American Joint Committee on Cancer (AJCC) Eighth Edition; no history of other tumors or other antitumor therapy; patients who received immunotherapy-based NAT with planned surgery; patients with at least 2 chest CT results available performed within 2 weeks (up to a maximum of 1 month) before immunotherapy-based NAT and 1 week (up to a maximum of half a month) before surgery.

Exclusion criteria include CT scans not done in our hospital or outside the study timeline (n = 11); Surgical cases done outside our hospital (n = 10).

Finally, the data of 62 patients were included in the study. They then were randomly divided into the training group (for the establishment of the radiomics label and model) and a validation group (for the verification of the radiomics label and model) at a ratio of 7:3 or 8:2.

### Pathology

#### Preoperative pathological information

The preoperative pathology was mainly determined by lung biopsy or bronchoscopy biopsy, and cases biopsied outside our hospital were all reconfirmed by our pathologists.

Preoperative pathologic information included common immunohistochemistry, genetic testing, and PD-L1 testing, Ki67 (percentage), chromogranin A (CgA) (negative/positive), Syn (negative/positive), p63 (strong/moderate/weak), cancer embroyonic antigen (CEA) (negative/positive), thyroid transcription factor 1 (TTF-1) (negative/positive), P40 (negative/positive), p53 (negative/positive), napsin-a (negative/positive), cytokeratin 5/6 (CK5/6) (negative/positive), cytokeratin 7 (CK7) (negative/positive), pan-cytokeratin (CK-Pan) (strong/moderate/weak), ALK control X3/echinoderm microtubule-associated protein-like 4 (EML4-ALK) (Ventana) (negative/positive), gene mutation (negative/positive), PD-L1 (22c3)-Ventana (total positive score) (negative/positive).

#### Postoperative pathological information

The postoperative pathology was confirmed from surgical resection samples after NAT immunotherapy, and the efficacy of NAT in NSCLC was evaluated by the pathologists of our hospital depending on tumor bed, lymph node tumor remission, and residual disease according to an expert consensus issued by the expert committee on lung cancer quality control at the National Cancer Quality Control Center (NCQCC) with the following criteria: MPR as viable tumor cell residual ≤10% from the tumor bed, CPR as no viable tumor cells remaining in the tumor bed and lymph nodes, and partial pathological response (PPR) as >10% viable tumor cells remaining in the tumor bed (14).

Collected postoperative pathology information, including PPR, MPR, and CPR, was defined as a good and poor pathological response (CPR, MPR) and poor pathological response (PPR).

### Patient clinical data

Clinical information was derived from the electronic medical record and was cross-checked by two independent investigators.

General clinical information collected includes age (years), gender (men/women), smoking status (yes/no), tumor differentiation grade (low/intermediate/well-differentiated), tumor type (squamous/adenocarcinoma), T stage (stage I/II/III/IV), N stage (stage I/II), clinical Tumor, Node, and Metastasis (TNM) stage (stage Ib–III), smoking history (yes/no), family history of tumor (yes/no), history of chronic comorbities (yes/no), height (m), number of immunotherapy cycles (two cycles/three cycles/four cycles), and immunodrugs (domestic/imported).

Clinical information before and after treatment was also collected, including body weight (kg), body mass index (kg/m^2^), lactate dehydrogenase (high/low), albumin (g/L), C-reactive protein (mg/L), white blood cells (10^9^/L), percentage of lymphocytes (%), tumor markers (normal/abnormal), thyroid function (normal/abnormal), T helper/induced T cells (%), and inhibitory T cells/cytotoxic T cells (%).

### Processing of missing data

The filling method for missing data is called data interpolation, which can be divided into the single filling and multiple filling methods. The single imputation method only yields one set of imputation results for an incomplete data set and greatly impacts the data distribution; for example, the mean filling method is to fill in missing values using the mean of all available data. The multiple imputation method uses the existing data of the incomplete data set to fill the missing value at any time to generate multiple complete data sets.

Random forest-based chained equations with multiple imputations (MICEforest, multiple imputations based on forest by chained equations) enable the generation of multiple groups of data with mean and variance that are all like the original data set according to the method of random forest, with imputation completed by comparing a selected group of data with the smallest difference from the original data set. Technically, any predictive model can be used for MICEforest.

This study deleted categories with missing values greater than 25%, and MICEforest multiple interpolation methods were used to fill the missing values. Category “thyroid function” was also removed because of the large difference between the data after filling and the original value.

### Processing of clinicopathological information

After deleting items with a lot of missing data and multiple imputing items with a small amount of missing data, the final remaining clinicopathological data used in this study were used to compare the distribution of variables in the two groups of good pathological response (GPR) vs. bad pathological response (BPR) using the R language program. The chi-square test was used for categorical variables, the Wilcoxon rank and t-test were used for continuous variables, and a p-value <0.05 was considered statistically significant.

### CT scan protocol

Patients were instructed to hold their breath after deep inspiration and complete the scan with one breath-hold using one of three CT scanners: Toshiba Aquilion ONE (Toshiba Medical System Corporation, Japan), Siemens SOMATOM Drive (Siemens Medical System Co., Ltd., Germany), and GE Revolution (General Electric Medical System Co., Ltd., America) with the scan ranging from at least the thoracic inlet to the level of the costophrenic angle, including the whole lung. Scanning parameters of different CT scanners are shown in **Table 1**.

**Table 1:** Scanning parameters of the different CT scanners.

	Toshiba Aquilion ONE	Siemens SOMATOM Drive	GE Revolution
tube current	automatic tube current	80-350mA automatic tube current	80-350mA automatic tube current
tube voltage	120 kV	120 kV	120 kV
FOV	320.3	307	\
construction algorithm	standard algorithm, lung algorithm and soft tissue algorithm	standard algorithm, lung algorithm and soft tissue algorithm	standard algorithm, lung algorithm and soft tissue algorithm
slice thickness	1mm	1mm	1mm
slice separation	0.8mm	1mm	1mm
matrix	512×512	512×512	512×512
construction slice separation	1mm	1mm	1
Construction slice thickness	1mm	1mm	1
revolution speed	\	\	158.75mm/s
detector width	\	\	80mm

FOV, field of view.

### Tumor segmentation

**Figure 1:**
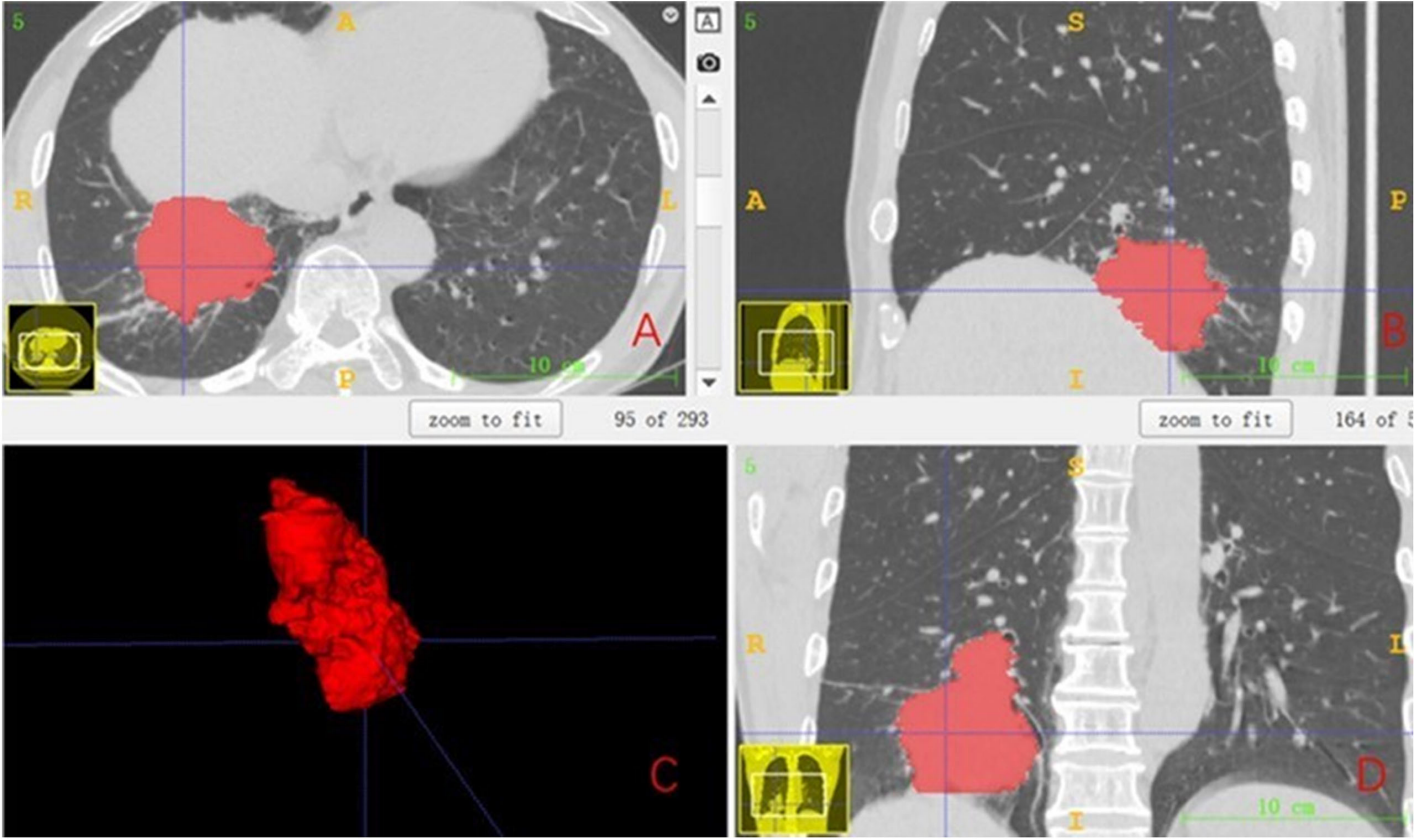
The final ROI. **(A)** Segmentation results of the cross section. **(B)** Segmentation results of the sagittal plane. **(C)** Three-dimensional (3D) visualization effect of the tumor area. **(D)** Segmentation results of the coronal plane. ROI, region of interest.

From the picture archiving and communication system (PACS), the CT images of each patient containing at least a lung window and mediastinal window were exported, and the enhanced CT was also exported synchronously if it existed. All cases were performed with the open-source software ITK‐SNAP (version 3.8.0, http://www.itk-snap.org) in high-resolution lung windows (window width 1,500–2,000 Hu, window position −450 to −600 Hu). In all CT images containing tumor lesions, the ROI was manually outlined along the contour of the lesion layer by layer to try to keep the ROI containing only the entire tumor and does not contain other distinguishable tissues, such as air and obvious blood vessels. If there is a simultaneously enhanced CT or PET, it will be compared layer by layer to make it as accurate as possible. The final ROI is shown in **Figure 1**.

### Radiomics conventional feature extraction and model construction

The manually drawn ROI was used to extract the traditional quantitative features of each patient using the Pyradiomics package (version 3.0.1, https://pyradiomics.readthedocs.io) in the Python program (version 3.6.13, https://www.python.org). The extracted radiomics traditional features included first-order features, two-dimensional (2D) shape features, 3D shape features, gray-level size zone matrix (GLSZM) features, gray-level co-occurrence matrix (GLCM) features, gray-level dependence matrix (GLDM) features, gray-level run-length matrix (GLRLM) features, and wavelet transform features. The extracted traditional features of radiomics and the clinicopathological information after deletion and imputation according to whether they were acquired before neoadjuvant immunotherapy or acquired after NAT were used to construct models separately. CT images acquired before NAT were extracted for radiomics; traditional quantitative features named before_rad_data, together with clinicopathological information that was available immediately before NAT, were used to construct the model, which is referred to as before_rad_cil. After 2–4 cycles of NAT, CT images obtained before surgery were used to extract radiomics quantitative features named after_rad_ data. Combined with the clinical information after NAT, a model named after_ rad_cil was constructed.

Before modeling, the data are transformed into structured data with 0 mean 1 variance by standardization for subsequent processing to eliminate the differences of different eigenvalues on the scale. After data normalization, features were screened by the Spearman correlation coefficient, and only one feature was retained in features with a high correlation (correlation coefficient >0.9).

**Figure 2:**
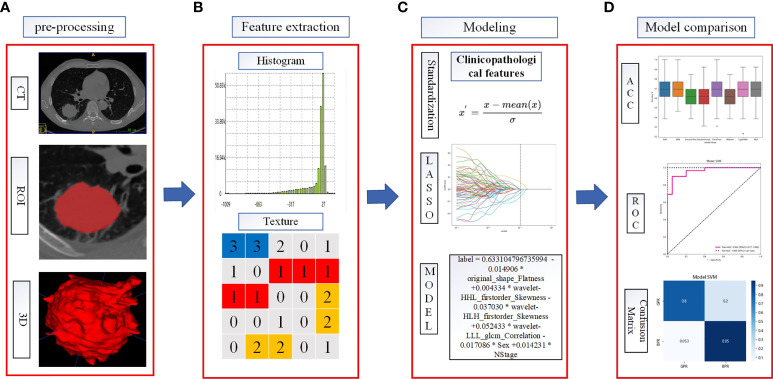
Flowchart of radiomics analysis. **(A)** The ROI was manually segmented on CT images before and after neoadjuvant therapy. **(B)** Quantitative features in the ROI were calculated, including features such as shape, texture, and wavelet filtering. **(C)** Combined clinicopathological features, standardized processing of data, feature filtering, and model building. **(D)** To evaluate the predictive model efficacy, the evaluation indexes of each model were compared to select the better performing model to construct the before_rad_cil_signature and after_rad_cil_signature. *multiply.

For the filtered features, the least absolute shrinkage and selection operator (LASSO) was used to select features to construct the LASSO equation and calculate the feature weights, respectively. These features with a feature coefficient >0 were randomly divided into training and test sets in a ratio of 7:3 and then modeled with one of eight machine learning algorithms: support vector machine (SVM), k-nearest neighbor (KNN), decision tree (DecisionTree), random forest (RandomForest), extreme gradient boosting (XGBoost), multilayer perceptron (MLP), extremely randomized trees (ExtraTrees), and light gradient boosting machine (LightGBM), And to compare the predictive accuracy of each model. The accuracy, area under the curve (AUC), sensitivity, and specificity of 5-fold cross-validation after random grouping were used as evaluation indexes. Finally, the model results with the best prediction efficiency after 100 random groupings were selected to construct the pretreatment radiomics features combined with the clinicopathological feature label (before_rad_cil_signature) and the posttreatment radiomics features combined with the clinicopathological feature label (after_rad_cil_signature). The flow of the ROI delineation, conventional feature extraction, data analysis, model building, and comparison is shown in **Figure 2**.

Tools used in combined clinicopathological feature and radiomics feature screening and model building were as follows: Python program (version 3.6.13, https://www.python.org/) and the packages of scikit-learn (version 1.0, https://scikit-learn.org) and pandas (version 1.1.5, https://pandas.pydata.org).

### Radiomics deep learning feature extraction and model construction

Usually, training a DL model requires large amounts of annotated data and an excellent performance hardware platform. Due to this study’s limited sample size and hardware platform, transfer learning and fine-tuning are used to overcome this limitation. Transfer learning can transfer knowledge learned from previous tasks to new tasks and avoid retraining new tasks to improve the learning efficiency of new tasks. Fine-tuning is usually used with transfer learning.

The pretrained model constructed by previous tasks is used to learn the task data to avoid the large amounts of human, computational, material, and financial resources required to retrain the model while ensuring its effectiveness. The pretrained model used in our study was resnet50, and the model network was pretrained on Imagenet to determine all network parameters through parameter adjustment and optimization.

**Figure 3:**
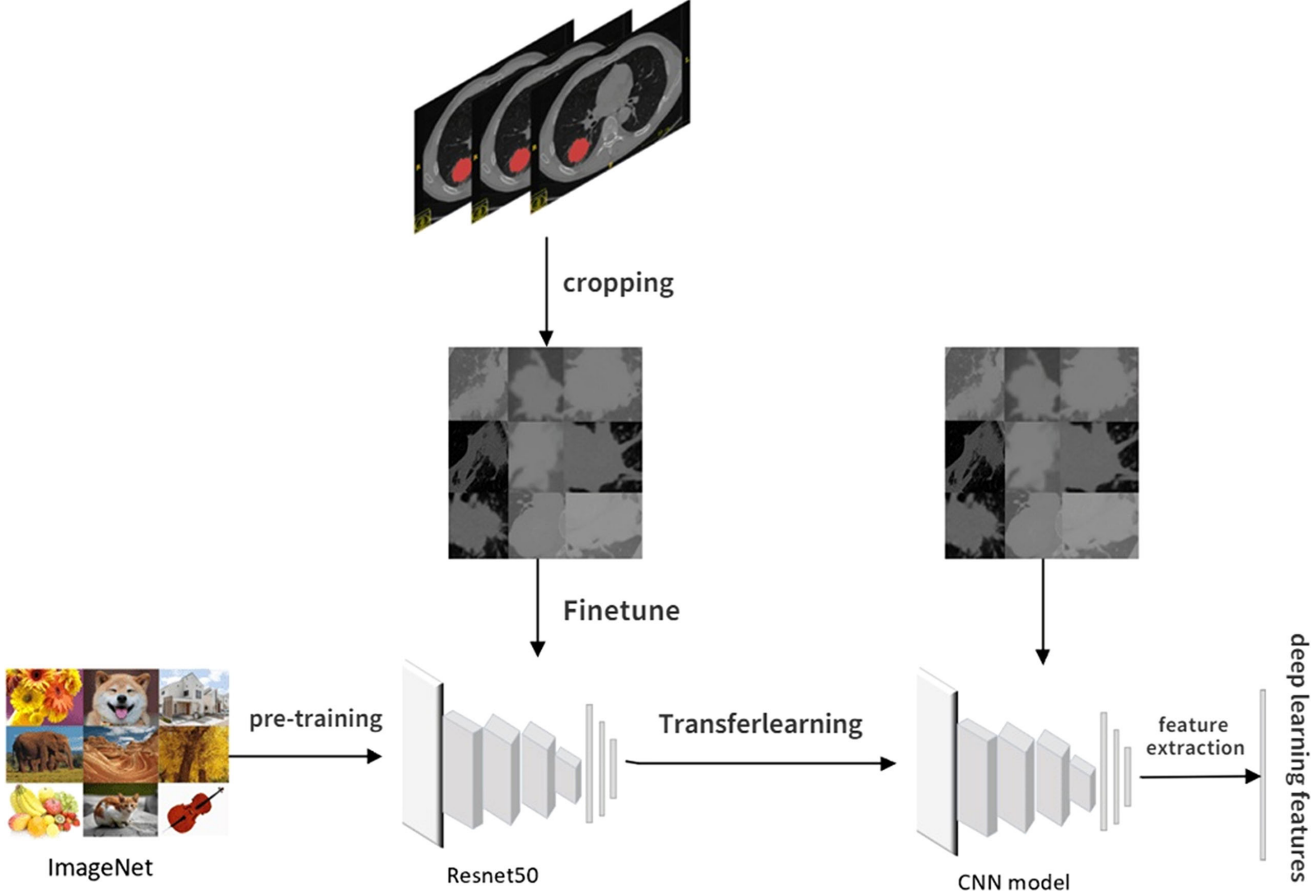
Flowchart of deep learning feature extraction.

The CT images for fine-tuning and DL feature extraction in this study were obtained by intercepting the tumor area at the layer of the maximum ROI on the cross section. The steps of DL feature extraction are shown in **Figure 3**: 1) Manually outline the ROI layer by layer using ITK-SNAP software. 2) The CT 3D image containing the ROI was automatically read using software written by ourselves, identifying the level at which the largest ROI was located in the cross section and clipping out the CT image at which the tumor was located with a rectangular box. 3) The cropped CT pictures were randomly split into training and validation groups in an 8:2 ratio, fine-tuned using the pretrained model resnet50. 4) Select the most accurate grouping model for DL feature extraction. 5) The output of DL features from the last layer before the full connection layer is usually the most concise. It is also the general DL feature selection layer in the industry. Therefore, our study selected the data of the last layer before the full connection layer, avgpool, as the DL features for subsequent studies, with a total of 2,048 features. 6) Like the above steps, the CT images obtained before and after NAT were separately subjected to DL feature extraction.

According to the CT images obtained before NAT, the extracted DL feature is named before_dl_data. According to the CT images obtained before surgery after 2–4 cycles of NAT, the DL feature is named after_dl_data.

Before modeling, the above data are converted into structured data with 0 mean 1 variance by standardization for subsequent processing. The data dimension is reduced to 62 dimensions by principal component analysis (PCA) after data standardization. The data set after dimension reduction was randomly divided into a training set and a test set in an 8:2 ratio. Then, this was modeled with one of eight machine learning algorithms: SVM, KNN, DecisionTree, RandomForest, XGBoost, MLP, ExtraTrees, and LightGBM, And to compare the predictive accuracy of each model. The accuracy, AUC, sensitivity, and specificity of 5-fold cross-validation after random grouping were used as evaluation indexes. Finally, the model results with the best prediction efficiency after 100 random groupings were selected to construct the pretreatment DL label (before_dl_signature) and the posttreatment DL label (after_dl_signature).

Tools used in DL feature extraction and model building were as follows: ITK‐SNAP (version 3.8.0, http://www.itk-snap.org), Python program (version 3.6.13, https://www.python.org), and packages: Pytorch (version 1.9.0, https://pytorch.org), scikit-learn (version 1.0, https://scikit-learn.org), and pandas (version 1.1.5, https://pandas.pydata.org).

### Combined model construction

After deletion and missing value processing, all previously extracted radiomics traditional quantitative features, radiomics DL features, and clinicopathological features were combined into a 7,421-dimensional joint data set. Before modeling, the joint data were transformed into structured data with 0 mean 1 variance by standardization to eliminate the scale difference of different eigenvalues. After data normalization, features were screened by the Spearman correlation coefficient, and only one was retained in features with a high correlation (correlation coefficient >0.9). LASSO further screens the selected features, and the LASSO equation is constructed for the features with feature coefficient >0, and the feature weights are calculated. These features with feature coefficient >0 were randomly divided into training and test sets in a ratio of 7:3. Then, these were modeled with one of eight machine learning algorithms: SVM, KNN, DecisionTree, RandomForest, XGBoost, MLP, ExtraTrees, LightGBM, And to compare the predictive accuracy of each model. The accuracy, AUC, sensitivity, and specificity of 5-fold cross-validation after random grouping were used as evaluation indexes. Finally, the model results with the best prediction efficiency after 100 random groupings were selected to construct the entire feature label (entire_signature).

Tools used in the entire feature screening and model building were as follows: Python program (version 3.6.13, https://www.python.org) and packages: scikit-learn (version 1.0, https://scikit-learn.org) and pandas (version 1.1.5, https://pandas.pydata.org).

### Nomogram construction

**Figure 4:**
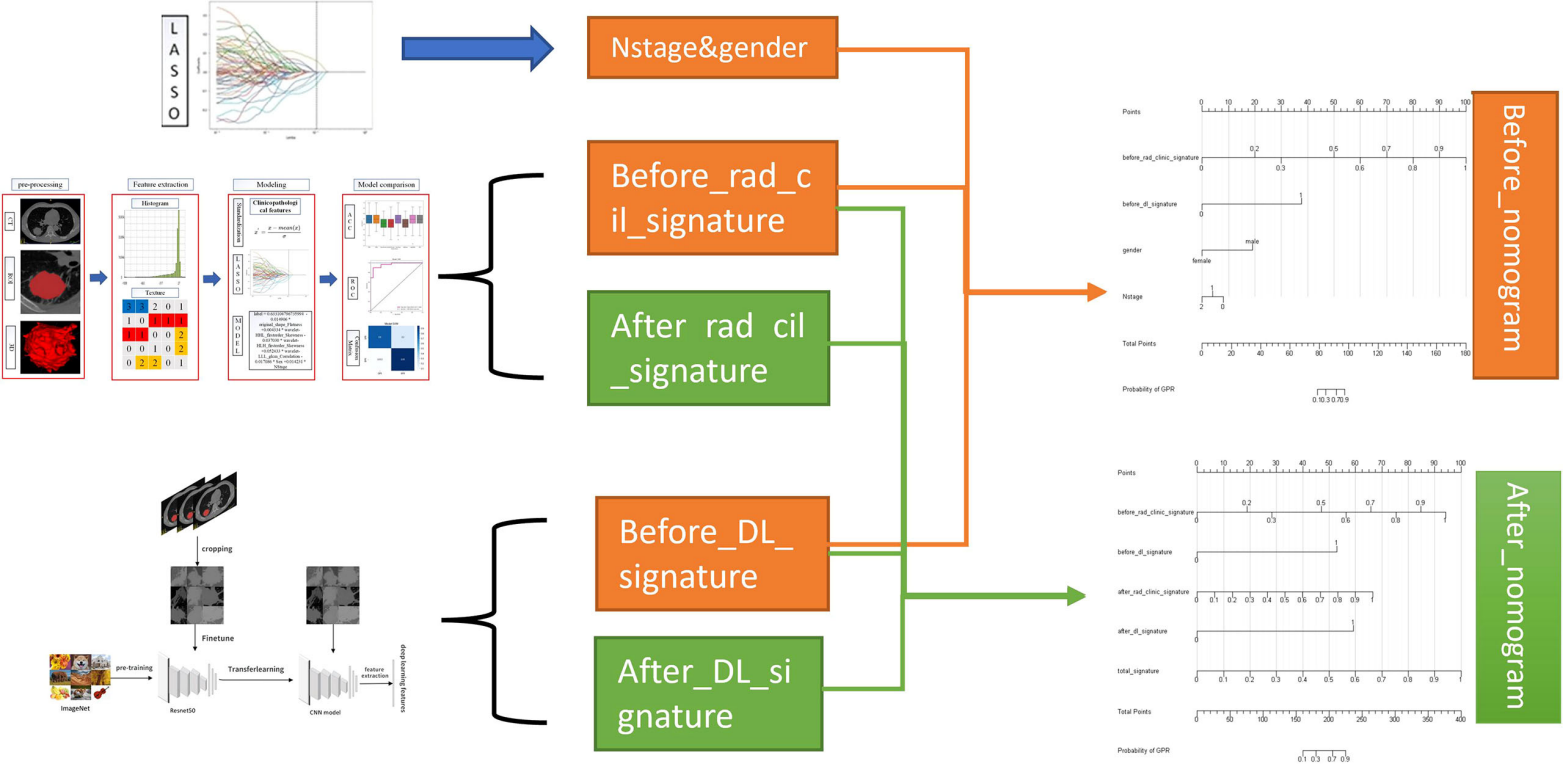
Nomogram construction flowchart.

The nomogram provides a simple graphical presentation of a clinical prediction model, allowing calculation of the probability of a certain target event based on individualized information for the patient. Its simple graphical interface promotes the wide application of nomograms. Our study used previously constructed before_rad_clinic_signature, before_dl_signature, and clinical features screened by LASSO to construct a pre-NAT nomogram (before_nomogram); before_rad_clinic_signature, before_dl_signature, after_rad_clinic_signature, after_dl_signature, and entire_signature were used to jointly construct a posttreatment nomogram. The procedure of nomogram construction is shown in **Figure 4**. Both nomograms were based on logistic regression models by plotting calibration curves to compare predicted vs. actual outcome events.

Tools used in nomogram construction were as follows: the R program (version 3.6.1, https://www.r-project.org) based on the RMS package (version 5.1-3.1).

## Results

### Clinicopathological information of patients

The categories, totals, and missing numbers of clinicopathological information collected are shown in **Tables 2** and **3**. The categories with missing values greater than 30% were CK, Caudal-related homeobox transcription factor-2 (CDX-2), CEA, p53, CD56, CgA, Syn, p63, CK-Pan, Ki67, CK7, PD-L1 expression, Driving gene mutation, p40, Napsin-A, CK5/6, TTF-1, after_assisted/induced T, after_inhibited/cytotoxic T, before_inhibited/cytotoxic T, and before_assisted/induced T. These categories were deleted.

**Table 2:** Categories with missing values, numbers missing, and proportions of missingness in the clinicopathological data collected.

Category	Numbers missing	Proportions of missingness	Category	Numbers missing	Proportions of missingness
CK	60	0.968	Napsin-A	36	0.581
CDX-2	60	0.968	CK5/6	36	0.581
CEA	59	0.952	TTF-1	31	0.5
P53	57	0.919	after _ assisted / induced T	30	0.484
CD56	50	0.806	after _ inhibited / cytotoxic T	30	0.484
CgA	50	0.806	before _ inhibited / cytotoxic T	28	0.452
Syn	49	0.79	before _ assisted / induced T	28	0.452
P63	48	0.774	before_CRP	15	0.242
CK-Pan	45	0.726	before_ thyroid function	12	0.194
Ki67	43	0.694	after_ thyroid function	7	0.113
CK7	43	0.694	after_CRP	4	0.065
PD-L1 expression	40	0.645	differentiation	4	0.065
Driving gene mutation	39	0.629	after tumor markers	3	0.048
P40	36	0.581	before_LDH	3	0.048

**Table 3:** Categories with no missing values in the collected clinicopathological data.

Category	Numbers missing	Proportions of missingness	Category	Numbers missing	Proportions of missingness
before tumor markers	0	0	before_ albumin	0	0
after_L%	0	0	before_BMI	0	0
after_WBC	0	0	before_ weight	0	0
after_ albumin	0	0	height	0	0
after_LDH	0	0	chronic history	0	0
after_ weight	0	0	gender	0	0
after_BMI	0	0	smoking status	0	0
number of immunotherapy cycles	0	0	Evaluation of postoperative	0	0
			clinical TNM stage	0	0
family history of tumor	0	0	N stage	0	0
before_L%	0	0	T stage	0	0
before_WBC	0	0	tumor type	0	0
Classification of immunodrugs	0	0	age	0	0

### Missing data processing

**Figure 5:**
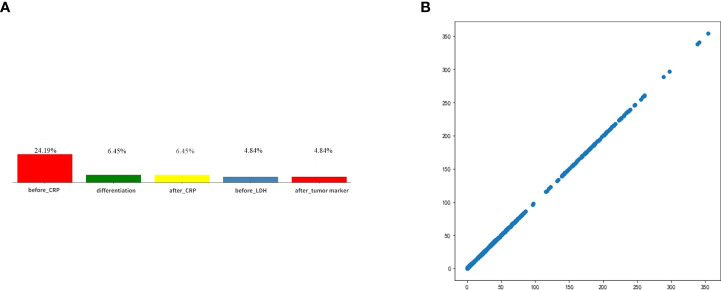
**(A)** Categories of missing values and ratios that need interpolation. **(B)** The fit of the imputed data to the original data.

After deleting the categories with missing values greater than 25%, the items and missing ratios that needed MICEforest for multiple interpolations are before_CRP, 24.19%; before_thyroid function, 19.35%; after_thyroid function, 11.29%; after_CRP, 6.45%; differentiation, 6.45%; after tumor markers, 4.84%; and before_LDH, 4.84%. Because the difference between the thyroid function data using multiple interpolations and the original data is more than 10%, they were deleted. The items and proportions of missing data that were ultimately included in the study and subjected to multiple imputations using the MICEforest method are shown in **Figure 5**. After multiple imputations, the mean difference between imputed and raw data was between 0.01% and 3%. **Figure 5** shows the fit of the imputed data to the original data.

### General information on clinical pathology

The distribution of clinicopathological information used for data analysis in this study was not statistically significant between GPR and BPR except for gender, as shown in **Table 4**.

**Table 4:** General information of clinicopathological information.

Category	Sub-classification	Total number	BPR	GPR	p-value	Test methods
		62	23	39		
gender	Women	11 (17.7%)	8 ( 34.8%)	3 ( 7.7%)	0.019	chi-square test
	Men	51 (82.3%)	15 ( 65.2%)	36 ( 92.3%)		
age(year)		58.5 (54.2-64.0)	58.0 (53.0-62.0)	59.0 (56.0-64.5)	0.321	Wilcoxon signed rank test
Type of tumor	adenocarcinoma	18 (29.0%)	10 ( 43.5%)	8 ( 20.5%)	0.102	chi-square test
	squamous	44 (71.0%)	13 ( 56.5%)	31 ( 79.5%)		
differentiation	intermediate	24 (38.7%)	12 ( 52.2%)	12 ( 30.8%)	0.226	chi-square test
	low	16 (25.8%)	4 ( 17.4%)	12 ( 30.8%)		
	well	22 (35.5%)	7 ( 30.4%)	15 ( 38.5%)		
T stage	III stage	16 (25.8%)	5 ( 21.7%)	11 ( 28.2%)	0.847	chi-square test
	II stage	26 (41.9%)	10 ( 43.5%)	16 ( 41.0%)		
	IV stage	16 (25.8%)	7 ( 30.4%)	9 ( 23.1%)		
	I stage	4 ( 6.5%)	1 ( 4.3%)	3 ( 7.7%)		
N stage	0 stage	16 (25.8%)	7 ( 30.4%)	9 ( 23.1%)	0.615	chi-square test
	II stage	32 (51.6%)	10 ( 43.5%)	22 ( 56.4%)		
	I stage	14 (22.6%)	6 ( 26.1%)	8 ( 20.5%)		
clinical stages	I/II stage	14 (22.6%)	6 ( 26.1%)	8 ( 20.5%)	0.721	chi-square test
	IIIA stage	34 (54.8%)	13 ( 56.5%)	21 ( 53.8%)		
	IIIB stage	14 (22.6%)	4 ( 17.4%)	10 ( 25.6%)		
smoking history	No	17 (27.4%)	9 ( 39.1%)	8 ( 20.5%)	0.196	chi-square test
	Yes	45 (72.6%)	14 ( 60.9%)	31 ( 79.5%)		
chronic history	No	48 (77.4%)	16 ( 69.6%)	32 ( 82.1%)	0.411	chi-square test
	Yes	14 (22.6%)	7 ( 30.4%)	7 ( 17.9%)		
height (m)		1.7 (1.6-1.7)	1.6 (1.6-1.7)	1.7 (1.6-1.7)	0.088	Wilcoxon signed rank test
before weight(kg)		61.2 (56.0-69.6)	60.0 (53.5-65.8)	64.0 (57.0-70.5)	0.117	Wilcoxon signed rank test
before_BMI(kg/m^2^)		22.6 (21.1-24.2)	22.6 (20.8-23.7)	22.7 (21.1-24.5)	0.6	Wilcoxon signed rank test
before_LDH(U/L)		175.1 (150.8-191.4)	178.5 (165.9-189.0)	175.0 (150.0-192.8)	0.925	Wilcoxon signed rank test
Before_albumin(g/L)		39.3 (37.2-41.6)	38.9 (36.9-41.5)	39.5 (37.3-41.5)	0.62	Wilcoxon signed rank test
before_CRP(mg/L)		9.5 (2.3-21.9)	7.0 (2.0-16.4)	10.4 (3.1-24.0)	0.166	Wilcoxon signed rank test
before_WBC(10^9/L)		6.7 (5.1-7.7)	5.7 (4.7-6.8)	7.0 (5.4-7.8)	0.022	Wilcoxon signed rank test
Before_lymphocyte percentage(%)		0.2 (0.2-0.3)	0.3 (0.2-0.3)	0.2 (0.2-0.3)	0.16	Wilcoxon signed rank test
Before_tumor markers	abnormal	34 (54.8%)	14 ( 60.9%)	20 ( 51.3%)	0.639	chi-square test
	normal	28 (45.2%)	9 ( 39.1%)	19 ( 48.7%)		
after_BMI(kg/m^2^)		23.2 (21.7-24.9)	22.8 (21.1-24.7)	23.6 (21.8-25.2)	0.336	Wilcoxon signed rank test
After_weight(kg)		64.0 (57.2-70.0)	60.0 (55.8-66.5)	65.5 (58.5-71.2)	0.085	Wilcoxon signed rank test
after_LDH(U/L)		199.2 (170.0-224.3)	204.0 (176.2-221.2)	193.0 (166.7-223.8)	0.359	Wilcoxon signed rank test
After_albumin(g/L)		40.0 (38.9-42.5)	41.4 (39.0-42.7)	40.0 (38.8-42.1)	0.517	Wilcoxon signed rank test
after_CRP(mg/L)		3.1 (2.2-5.7)	4.2 (2.1-6.6)	2.9 (2.3-5.2)	0.503	Wilcoxon signed rank test
after_WBC(10^9^/L)		4.7 (3.9-5.6)	4.7 (4.0-5.3)	4.6 (4.0-5.8)	0.793	Wilcoxon signed rank test
after_lymphocyte percentage(%)		0.3 (0.2-0.4)	0.3 (0.2-0.4)	0.3 (0.3-0.4)	0.431	Wilcoxon signed rank test
after_tumor markers	abnormal	13 (21.0%)	7 ( 30.4%)	6 ( 15.4%)	0.279	chi-square test
	normal	49 (79.0%)	16 ( 69.6%)	33 ( 84.6%)		
number of immunotherapy cycles	two cycles	5 ( 8.1%)	2 ( 8.7%)	3 ( 7.7%)	0.982	chi-square test
	three cycles	54 (87.1%)	20 ( 87.0%)	34 ( 87.2%)		
	four cycles	3 ( 4.8%)	1 ( 4.3%)	2 ( 5.1%)		
Classification of immunodrugs	domestic	28 (45.2%)	11 ( 47.8%)	17 ( 43.6%)	0.952	chi-square test
	imported	34 (54.8%)	12 ( 52.2%)	22 ( 56.4%)		
history of tumor	no	60 (96.8%)	23 (100.0%)	37 ( 94.9%)	0.719	chi-square test
	yes	2 ( 3.2%)	0 ( 0.0%)	2 ( 5.1%)		

### Radiomics conventional features and model building

#### Before_rad_data extraction and before_rad_cil model construction

Python Pyradiomics package extracted radiomics Conventional quantitative features from CT images, extracting 1,648 features per patient. Features extracted from all CT images before NAT were constructed as the before_rad_data. There were 197 first-order features (First order), 13 2D shape features, 231 3D shape features, 242 GLCM features, 84 GLDM features, 96 GLRLM features, 96 GLSZM features, and 688 wavelet transform features in the before_rad_data.

**Figure 6:**
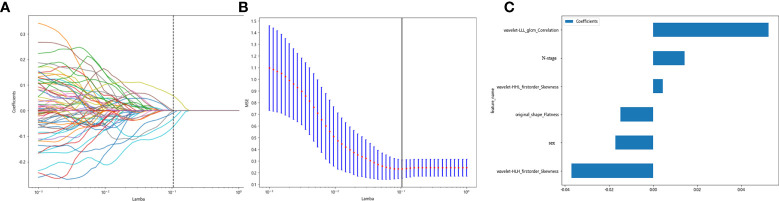
Regression feature screening. **(A)** Feature selection plot for the LASSO regression, which was adjusted by a super parameter (Lamba), to achieve the purpose of screening the optimal features. The vertical dashed line indicates that the corresponding optimal Lamba value when obtaining the minimum deviation value is Lamba = 0.1048. **(B)** The convergence graph of characteristic coefficients for feature selection by cross-validation. Features with non-zero coefficients were screened out corresponding to the vertical lines in the plot, with a total of six best features selected. **(C)** Features and weights of LASSO regression screening. The six features screened by LASSO were “original_shape_Flatness,” “wavelet-HHL_firstorder_Skewness,” “wavelet-HLH_firstorder_Skewness,” “wavelet-LLL_glcm_Correlation,” “gender,” and “N_stage”.

Clinicopathological characteristics before neoadjuvant treatment are shown in **Table 5**, used with the before_rad_data, and jointly constructed into a 2D array with a feature number of 1,667. After screening by the Spearman correlation coefficient, 238 features related to the efficacy of neoadjuvant immunotherapy were obtained. The screened features were randomly divided into training and test groups in a 7:3 ratio and further screened by the LASSO regression model. At Lamba = 0.1048 (**Figure 6**), six features highly correlated with the efficacy of neoadjuvant immunotherapy (four radiomics traditional features and two clinical features) were obtained, and the selected six features were combined into one label using a generalized linear model, and the label score was calculated for each patient. The label score is calculated as follows: label score = 0.633104796735994 - 0.014906 * original_shape_Flatness + 0.004334 * wavelet-HHL_firstorder_Skewness - 0.037030 * wavelet-HLH_firstorder_Skewness + 0.052433 * wavelet-LLL_glcm_Correlation - 0.017086 * gender + 0.014231 * N_stage.

**Table 5:** Clinicopathological information converged with the before_rad_data.

Gender (men/women)	Age (years)	Tumor type (squamous / adenocarcinoma)	Differentiation grade (low/intermediate/well differentiated)
smoking history (yes / no)	history of tumor (yes / no)	chronic history (yes / no)	beforeweight(kg)
before_LDH(U/L)	before albumin(g/L)	before_CRP(mg/L)	before_WBC(10^9/L)
T stage(I/II/III/IVstage)	N stage(I/II stage)	Clinical TNM stage(IB-III stage)	before tumor markers (normal / abnormal)
before_BMI(kg/m^2^)	before_ percentage of lymphocytes(%)	height(m)	

**Figure 7:**
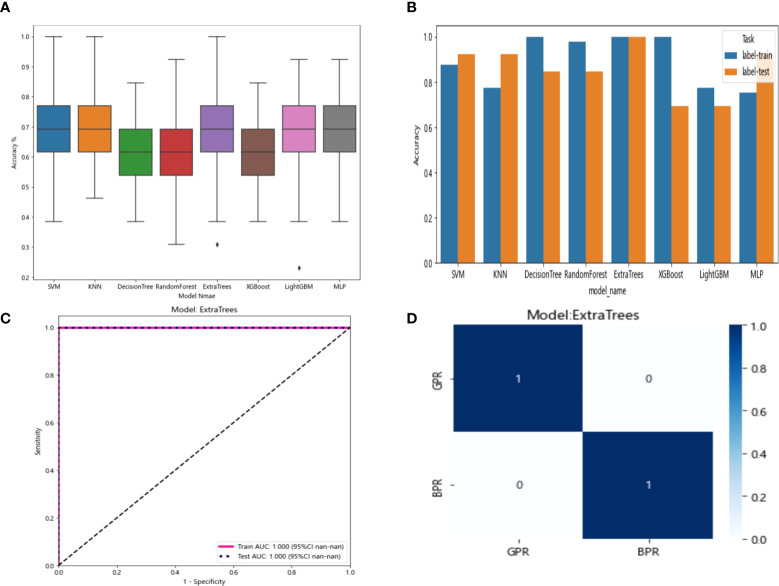
**(A)** Distribution of the accuracy of each model randomly tested 100 times by eight common machine learning algorithms. **(B)** Comparison of the accuracy of the best performing one out of 100 randomizations in eight machine learning algorithms. **(C)** AUCs of the training set and test set in the Extratrees model. **(D)** Confusion matrix for the Extratrees model.

The six features screened by LASSO regression are randomly divided into a training group and a test group according to the 7:3 ratio. Then, eight common machine learning algorithms are used to model. The accuracy of the model obtained by 100 random grouping modeling is shown in **Figure 7** and **Table 6**. Three of the eight models (SVM, KNN, ExtraTrees) had a maximum accuracy of 1. The mean accuracy of all models was 0.708–0.599.

**Table 6:** Distribution of the accuracy of each model randomly tested 100 times by eight common machine learning algorithms.

model Names	SVM	KNN	DecisionTree	RandomForest	ExtraTrees	XGBoost	LightGBM	MLP
Number of tests	100	100	100	100	100	100	100	100
Mean accuracy	0.668	0.708	0.599	0.631	0.667	0.621	0.664	0.686
25 % quantile accuracy	0.615	0.615	0.538	0.538	0.615	0.538	0.615	0.615
50% quantile accuracy	0.692	0.692	0.615	0.615	0.692	0.615	0.692	0.692
75% quantile accuracy	0.769	0.769	0.692	0.692	0.769	0.692	0.769	0.769
Maximum accuracy	1	1	0.846	0.923	1	0.846	0.923	0.923

In the 100 random groupings, the performances of the best grouping in each model are shown in **Figure 7** and **Table 7**. The best model was Extratrees. The accuracy, AUC, sensitivity, and specificity in both training and test sets were 1. So, Extratrees model data were selected to build the before_rad_cil_signature for subsequent research.

**Table 7:** Each evaluation index of the best performance of 100 random groupings in eight machine learning algorithms.

Model names	Accuracy	AUC	Sensitivity	Specificity	Sets
SVM	0.878	0.962	0.897	0.95	train
SVM	0.923	1	1	1	test
KNN	0.776	0.863	0.828	0.7	train
KNN	0.923	0.95	0.9	1	test
DecisionTree	1	1	1	1	train
DecisionTree	0.846	0.9	0.8	1	test
RandomForest	0.980	0.997	0.966	1	train
RandomForest	0.846	0.883	0.8	1	test
ExtraTrees	1	1	1	1	train
ExtraTrees	1	1	1	1	test
XGBoost	1	1	1	1	train
XGBoost	0.692	0.9	0.7	1	test
LightGBM	0.776	0.803	0.862	0.7	train
LightGBM	0.692	0.833	1	0.667	test
MLP	0.755	0.916	0.931	0.75	train
MLP	0.923	0.833	1	0.667	test

#### After_rad_data extraction and after_rad_cil model construction

The extraction method for the after_rad_data was consistent with the before_rad_data, and the data for extracting quantitative features were derived from CT images of all patients after NAT. Finally, the resulting quantitative feature categories and numbers are consistent with the before_rad_data.

**Figure 8:**
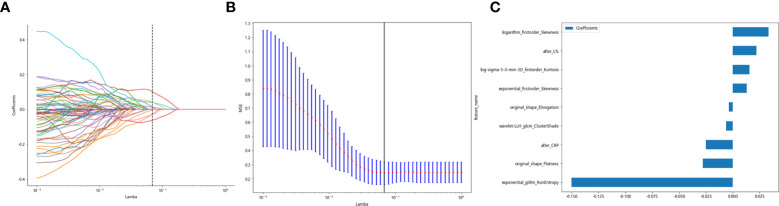
Regression feature screening. **(A)** Feature selection plot for the LASSO regression, which was adjusted by a super parameter (Lamba), to achieve the purpose of screening the optimal features. The vertical dashed line indicates that the corresponding optimal Lamba value when obtaining the minimum deviation value is Lamba = 0.06866. **(B)** The convergence graph of characteristic coefficients for feature selection by cross-validation. Features with non-zero coefficients were screened out corresponding to the vertical lines in the plot, with a total of nine best features selected. **(C)** Twelve features and weights of LASSO regression screening. The nine features screened were “exponential_firstorder_Skewness,” “exponential_glrlm_RunEntropy,” “log-sigma-5-0-mm-3D_firstorder_Kurtosis,” “logarithm_firstorder_Skewness,” “original_shape_Elongation,” “original_shape_Flatness,” “wavelet-LLH_glcm_ClusterShade,” “after_CRP,” and “after_percentage of lymphocytes”.

Clinicopathological characteristics after neoadjuvant treatment are shown in **Table 8**, fused with the after_rad_data and jointly constructed into a 2D array with a feature number of 1,658. After screening by the Spearman correlation coefficient, 237 features related to the efficacy of neoadjuvant immunotherapy were obtained. The screened features were randomly divided into training and testing groups in a 7:3 ratio, further screened by the LASSO regression model. At Lamba = 0.06866 (**Figure 8**), nine features highly correlated with the efficacy of neoadjuvant immunotherapy (seven radiomics traditional features and two clinical features) were obtained. The selected nine features were combined into one label using a generalized linear model, and the label score was calculated for each patient. The label score is calculated as follows: Label score = 0.6110867308836403 + 0.012900 * exponential_firstorder_Skewness - 0.150077 * exponential_glrlm_RunEntropy + 0.015404 * log-sigma-5-0-mm-3D_firstorder_Kurtosis + 0.033240 * logarithm_firstorder_Skewness - 0.003723 * original_shape_Elongation - 0.027763 * original_shape_Flatness - 0.006209 * wavelet-LLH_glcm_ClusterShade - 0.025090 * after_CRP + 0.022022 * after_percentage of lymphocytes.

**Table 8:** Clinicopathological information converged with the before_rad_data.

After_BMI(Kg/m2)	After_weight(kg)	After_LDH(U/L)	After_albumin(g/L)
after_CRP(mg/L)	after_WBC(10^9/L)	After_ percentage of lymphocytes(%)	After_ tumor markers (normal / abnormal)
number of immunotherapy cycles (two cycles / three cycles / four cycles)	Classification of immunodrugs (domestic / imported)		

**Figure 9:**
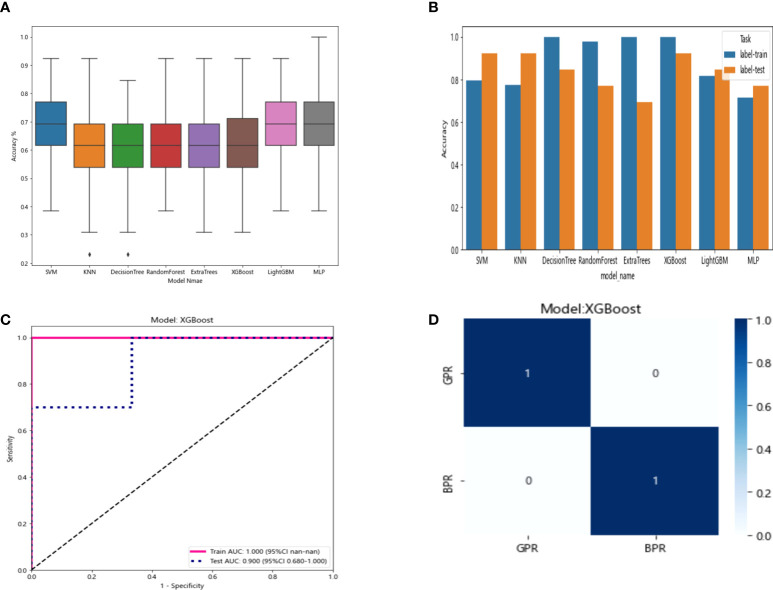
**(A)** Distribution of the accuracy of each model randomly tested 100 times by eight common machine learning algorithms. **(B)** Comparison of the accuracy of the best performing one out of 100 randomizations in eight machine learning algorithms. **(C)** AUCs of the training set and test set in the XGBoost model. **(D)** Confusion matrix for the XGBoost model.

The nine features screened by LASSO regression were randomly divided into a training group and a testing group according to the ratio of 7:3. Then, eight common machine learning algorithms are used to model. The accuracy of the model obtained by 100 random grouping modeling is shown in **Figure 9** and **Table 9**. One of the eight models (MLP) had a maximum accuracy of 1. The maximum accuracy of the other models ranged from 0.846 to 0.923. The mean accuracy of all models was 0.602–0.682.

**Table 9:** Distribution of the accuracy of each model randomly tested 100 times by eight common machine learning algorithms.

Model Names	SVM	KNN	DecisionTree	RandomForest	ExtraTrees	XGBoost	LightGBM	MLP
Number of tests	100	100	100	100	100	100	100	100
Mean accuracy	0.682	0.602	0.611	0.627	0.637	0.645	0.675	0.68
25 % quantile accuracy	0.616	0.538	0.538	0.538	0.538	0.538	0.615	0.615
50% quantile accuracy	0.692	0.615	0.615	0.615	0.615	0.615	0.692	0.692
75% quantile accuracy	0.769	0.692	0.692	0.692	0.692	0.712	0.769	0.769
Maximum accuracy	0.923	0.923	0.846	0.923	0.923	0.923	0.923	1

In the 100 random groupings, the performances of the best grouping in each model are shown in **Figure 9** and **Table 10**. The best model was XGBoost. The accuracy, AUC, sensitivity, and specificity in the training set were 1 and in the testing set were 0.923, 0.9, 0.7, and 1, respectively. So, XGBoost model data were selected to build the after_rad_cil_signature for subsequent research.

**Table 10:** Each evaluation index of the best performance of 100 random groupings in eight machine learning algorithms.

Model names	Accuracy	AUC	Sensitivity	Specificity	Sets
SVM	0.796	0.928	0.828	0.9	train
SVM	0.923	0.967	0.9	1	test
KNN	0.776	0.8	0.793	0.75	train
KNN	0.923	0.967	0.8	1	test
DecisionTree	1	1	1	1	train
DecisionTree	0.846	0.783	0.9	1	test
RandomForest	0.980	0.999	0.966	1	train
RandomForest	0.769	0.833	0.5	1	test
ExtraTrees	1	1	1	1	train
ExtraTrees	0.692	0.8	0.5	1	test
XGBoost	1	1	1	1	train
XGBoost	0.923	0.9	0.7	1	test
LightGBM	0.816	0.872	0.862	0.85	train
LightGBM	0.846	0.833	1	0.667	test
MLP	0.714	0.910	0.862	0.9	train
MLP	0.769	1	1	1	test

### Radiomics deep learning model building

#### Before_dl model building

**Figure 10:**
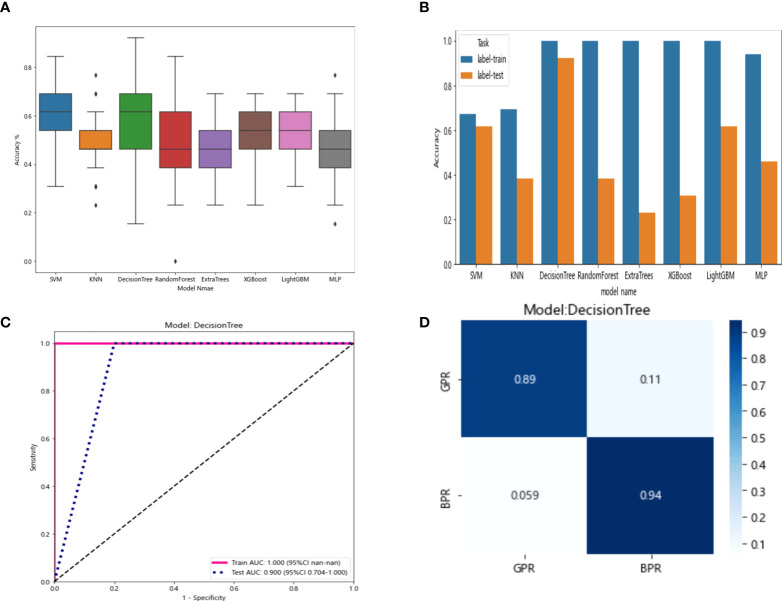
**(A)** Each model’s accuracy distribution was randomly tested 100 times by eight common machine learning algorithms. **(B)** Comparison of the accuracy of the best performing one out of 100 randomizations in eight machine learning algorithms. **(C)** AUCs of the training set and test set in the DecisionTree model. **(D)** Confusion matrix for the DecisionTree model.

The resnet50 DL model after fine-tuning was used to extract features, obtaining 2,048-dimensional DL features from all CT images obtained before NAT by intercepting the lung window images at the maximum level of the tumor in the cross section. The 62-dimensional data set was obtained by PCA dimensionality reduction and randomly divided into training and testing groups according to the ratio of 8:2. Then, eight common machine learning algorithms were used to model. The accuracy of the model obtained by 100 random grouping modeling is shown in **Figure 10** and **Table 11**. One of the eight models (DecisionTree) had a maximum accuracy of 0.923. The mean accuracy of all models was 0.469–0.629.

**Table 11:** Distribution of the accuracy of each model randomly tested 100 times by eight common machine learning algorithms.

model Names	SVM	KNN	DecisionTree	RandomForest	ExtraTrees	XGBoost	LightGBM	MLP
Number of tests	100	100	100	100	100	100	100	100
Mean accuracy	0.629	0.492	0.599	0.489	0.469	0.511	0.515	0.472
25 % quantile accuracy	0.538	0.462	0.462	0.385	0.385	0.462	0.462	0.385
50% quantile accuracy	0.615	0.462	0.615	0.462	0.462	0.538	0.538	0.462
75% quantile accuracy	0.692	0.538	0.692	0.615	0.538	0.615	0.615	0.538
Maximum accuracy	0.846	0.769	0.923	0.846	0.692	0.692	0.692	0.769

In the 100 random groupings, the performances of the best grouping in each model were shown in **Figure 10** and **Table 12**. The best model was DecisionTree. The accuracy, AUC, sensitivity, and specificity in the training set were 1 and in the testing set were 0.923, 0.9, 1, and 1. So, DecisionTree model data were selected to build the before_dl_signature for subsequent research.

**Table 12:** Each evaluation index of the best performance of 100 random groupings in eight machine learning algorithms.

Model names	Accuracy	AUC	Sensitivity	Specificity	Sets
SVM	0.673	0.113	0.968	0.059	train
SVM	0.615	0.375	0.5	0.75	test
KNN	0.694	0.659	0.871	0.5	train
KNN	0.385	0.263	1	0	test
DecisionTree	1	1	1	1	train
DecisionTree	0.923	0.9	1	1	test
RandomForest	1	1	1	1	train
RandomForest	0.385	0.425	0.5	0.75	test
ExtraTrees	1	1	1	1	train
ExtraTrees	0.231	0.063	1	0	test
XGBoost	1	1	1	1	train
XGBoost	0.308	0.35	0.5	0.75	test
LightGBM	1	1	1	1	train
LightGBM	0.615	0.2	1	0	test
MLP	0.939	0.996	0.968	1	train
MLP	0.462	0.325	0.125	1	test

#### After_dl model building

**Figure 11:**
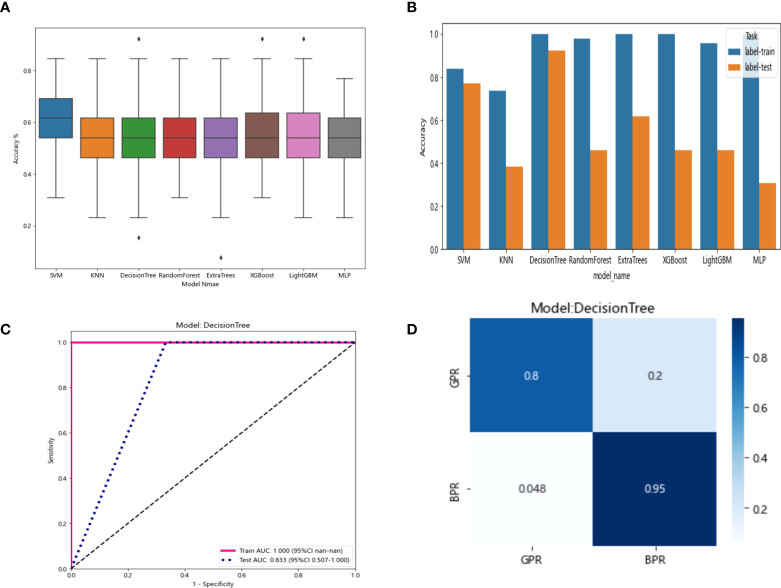
**(A)** Each model’s accuracy distribution was randomly tested 100 times by eight common machine learning algorithms. **(B)** Comparison of the accuracy of the best performing one out of 100 randomizations in eight machine learning algorithms. **(C)** AUCs of the training set and test set in the DecisionTree model. **(D)** Confusion matrix for the DecisionTree model.

The resnet50 DL model after fine-tuning was used to extract features, obtaining 2,048-dimensional DL features from all CT images obtained after NAT by intercepting the lung window images at the maximum level of the tumor in the cross section. The 62-dimensional data set was obtained by PCA dimensionality reduction and randomly divided into training and testing groups according to the ratio of 8:2. Then, eight common machine learning algorithms were used to model. The accuracy of the model obtained by 100 random grouping modeling is shown in **Figure 11** and **Table 13**. Three of the eight models (DecisionTree, XGBoost, and LightGBM) had a maximum accuracy of 0.923. The mean accuracy of all models was 0.509–0.603.

**Table 13:** Distribution of the accuracy of each model randomly tested 100 times by eight common machine learning algorithms.

model Names	SVM	KNN	DecisionTree	RandomForest	ExtraTrees	XGBoost	LightGBM	MLP
Number of tests	100	100	100	100	100	100	100	100
Mean accuracy	0.603	0.545	0.548	0.547	0.545	0.567	0.568	0.509
25 % quantile accuracy	0.538	0.462	0.462	0.462	0.462	0.462	0.462	0.462
50% quantile accuracy	0.615	0.538	0.538	0.538	0.538	0.538	0.538	0.538
75% quantile accuracy	0.692	0.615	0.615	0.615	0.615	0.635	0.635	0.615
Maximum accuracy	0.846	0.846	0.923	0.846	0.846	0.923	0.923	0.769

In the 100 random groupings, the performances of the best grouping in each model are shown in **Figure 11** and **Table 14**. The best model was DecisionTree. The accuracy, AUC, sensitivity, and specificity in the training set were 1 and in the testing set were 0.923, 0.833, 1, and 1. So, DecisionTree model data were selected to build the after_dl_signature for subsequent research.

**Table 14:** Each evaluation index of the best performance of 100 random groupings in eight machine learning algorithms.

Model names	Accuracy	AUC	Sensitivity	Specificity	Sets
SVM	0.837	0.017	1	0	train
SVM	0.769	0.733	0.6	1	test
KNN	0.735	0.769	0.793	0.65	train
KNN	0.385	0.25	1	NaN	test
DecisionTree	1	1	1	1	train
DecisionTree	0.923	0.833	1	1	test
RandomForest	0.980	0.999	0.966	1	train
RandomForest	0.462	0.45	0.5	0.667	test
ExtraTrees	1	1	1	1	train
ExtraTrees	0.615	0.367	1	0	test
XGBoost	1	1	1	1	train
XGBoost	0.462	0.433	0.4	1	test
LightGBM	0.959	0.984	0.966	1	train
LightGBM	0.462	0.433	0.4	1	test
MLP	1	1	1	1	train
MLP	0.308	0.033	1	0	test

### Entire model

**Figure 12:**
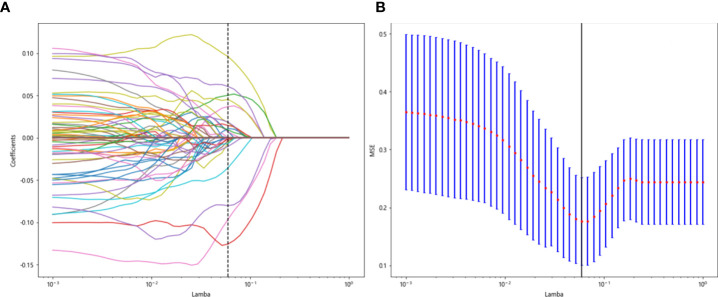
Regression feature screening. **(A)** Feature selection plot for the LASSO regression, which was adjusted by a super parameter (Lamba) to achieve the purpose of screening the optimal features. The vertical dashed line indicates that the corresponding optimal Lamba value when obtaining the minimum deviation value is Lamba = 0.0596. **(B)** The convergence graph of characteristic coefficients for feature selection by cross-validation. Features with non-zero coefficients were screened out corresponding to the vertical lines in the plot, with a total of 20 best features selected.

Note: “_before” represents the radiomics features before treatment, and “_after” represents the radiomics features after treatment. Naming is like “exponential_gldm_dependenceenterprise_before” for radiomics traditional features, and the naming is like “80_after” for DL features.

All previously extracted radiomics traditional quantitative features, radiomics DL features, and clinicopathological features after deletion and missing value processing were combined into a 7,421-dimensional joint data set. The combined data set obtained 4,266 characteristics related to the efficacy of immune NAT after standardization and Spearman correlation coefficient screening. The screened features were randomly divided into training and test groups in a 7:3 ratio, which was further screened by the LASSO regression model. At Lamba = 0.0596 (**Figure 12**), 20 features highly correlated with the efficacy of immune NAT (six radiomics traditional features, 11 DL features, and three clinical features) were obtained, and the selected 20 features were combined into one label using generalized linear model, and the label score was calculated for each patient. The label score is calculated as follows: Label score = 0.6143861851029497 + 0.003364 * exponential_gldm_DependenceEntropy_before - 0.045913 * wavelet-HLH_firstorder_Skewness_before - 0.008996 * wavelet-HLH_glcm_Correlation_before + 0.058934 * wavelet-LLL_glcm_Correlation_before + 0.028862 * square_glszm_SmallAreaLowGrayLevelEmphasis_after - 0.001464 * wavelet-LHL_glcm_ClusterShade_after - 0.051536 * gender + 0.002784 * age + 0.011369 * N_stage + 0.021636 * 642_before + 0.009017 * 61_after + 0.122782 * 80_after - 0.138473 * 199_after +0.021755 * 284_after + 0.036575 * 802_after - 0.008443 * 965_after + 0.038793 * 1508_after +0.034689 * 1538_after + 0.045343 * 1553_after + 0.000306 * 2030_after.

**Figure 13:**
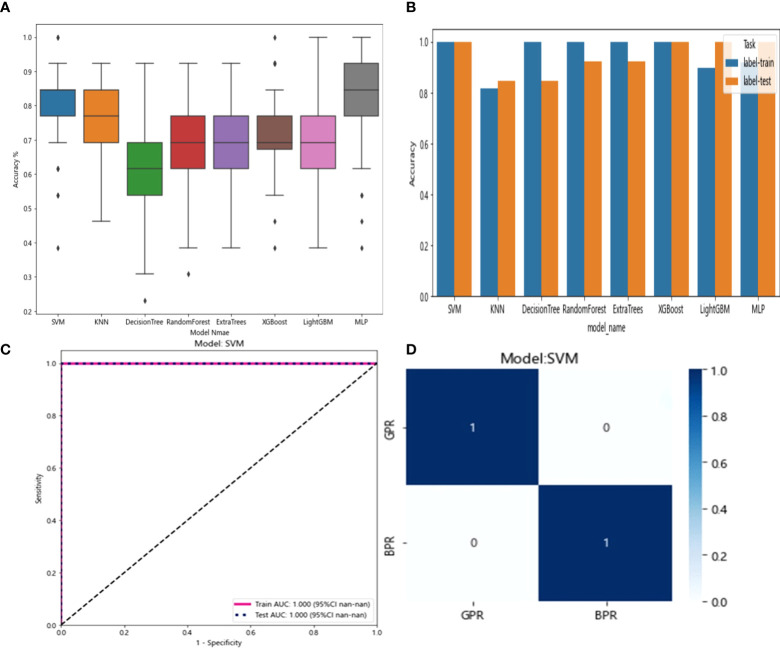
**(A)** Distribution of the accuracy of each model randomly tested 100 times by eight common machine learning algorithms. **(B)** Comparison of the accuracy of the best performing one out of 100 randomizations in eight machine learning algorithms. **(C)** AUCs of the training set and test set in the SVM model. **(D)** Confusion matrix for the SVM model.

The 20 features screened by LASSO regression are randomly divided into a training group and a testing group according to the ratio of 7:3. Then, eight common machine learning algorithms are used to model. The accuracy of the model obtained by 100 random grouping modeling is shown in **Figure 13** and **Table 15**. Four of the eight models (SVM, XGBoost, LightGBM, and MLP) had a maximum accuracy of 1. The mean accuracy of all models was 0.628–0.805.

**Table 15:** Distribution of the accuracy of each model randomly tested 100 times by eight common machine learning algorithms.

model Names	SVM	KNN	DecisionTree	RandomForest	ExtraTrees	XGBoost	LightGBM	MLP
Number of tests	100	100	100	100	100	100	100	100
Mean accuracy	0.801	0.732	0.628	0.714	0.714	0.723	0.702	0.805
25 % quantile accuracy	0.769	0.692	0.538	0.615	0.615	0.673	0.615	0.769
50% quantile accuracy	0.846	0.769	0.615	0.692	0.692	0.692	0.692	0.846
75% quantile accuracy	0.846	0.846	0.692	0.769	0.769	0.769	0.769	0.923
Maximum accuracy	1	0.923	0.923	0.923	0.923	1	1	1

In the 100 random groupings, the performances of the best grouping in each model are shown in **Figure 13** and **Table 16**. The best model was SVM and XGBoost. The accuracy, AUC, sensitivity, and specificity in both training and test sets were 1. However, the average accuracy of model SVM in 100 random grouping tests is higher. So, SVM model data were selected to build the entire_signature for further research.

**Table 16:** Each evaluation index of the best performance of 100 random groupings in eight machine learning algorithms.

Model names	Accuracy	AUC	Sensitivity	Specificity	Sets
SVM	1	1	1	1	train
SVM	1	1	1	1	test
KNN	0.816	0.872	0.821	0.810	train
KNN	0.846	0.977	0.909	1	test
DecisionTree	1	1	1	1	train
DecisionTree	0.846	0.705	0.909	1	test
RandomForest	1	1	1	1	train
RandomForest	0.923	1	1	1	test
ExtraTrees	1	1	1	1	train
ExtraTrees	0.923	0.932	0.909	1	test
XGBoost	1	1	1	1	train
XGBoost	1	1	1	1	test
LightGBM	0.898	0.927	0.929	0.857	train
LightGBM	1	1	1	1	test
MLP	0.918	0.986	0.929	0.952	train
MLP	1	1	1	1	test

### Nomogram

#### Drawing and calibration of the before_nomogram

**Figure 14:**
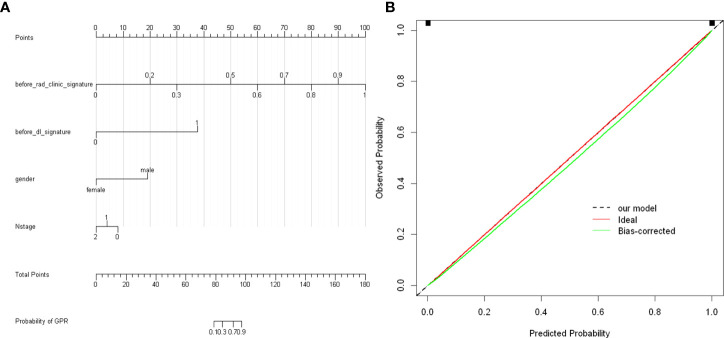
**(A)** before_nomogram.Locate on the before_rad_clinic_signature, before_dl_signature, gender, and N stage coordinate axis. Draw a straight line perpendicular to the first points, calculate and sum the scores corresponding to each straight line, locate on the total points coordinate axis, and draw a straight line perpendicular to the horizontal axis of the probability of GPR. The corresponding value is the probability of pathological response to GPR in patients with non-small cell lung cancer after neoadjuvant immunotherapy. **(B)** Calibration curve corresponding to the before_nomogram. The consistency between the probability of GPR predicted by the nomogram after 1,000 repeated samplings and the actual GPR. The 45° red line represents the ideal prediction performance. The black dotted line and the solid green line represent the prediction performance of the nomogram and the correction of the deviation of the nomogram, respectively. The closer the black dotted line is to the 45° ideal red line, the higher the model’s prediction accuracy.

To provide a simple graphical presentation of a clinical prediction model, our study used previously constructed before_rad_clinic_signature, before_dl_signature, and clinical features screened by LASSO to construct a pre-NAT nomogram (before_nomogram). According to the characteristics in **Figure 14A**, a patient’s corresponding probability of GPR after treatment can be calculated. **Table 17** shows the features in the before_nomogram and the corresponding scores. **Table 18** shows the GPR probability corresponding to different total scores in the before_nomogram. **Figure 14B** shows that the probability of GPR predicted by the nomogram after 1,000 repeated samplings and the actual GPR is consistent.

**Table 17:** The features in the nomogram and the corresponding scores.

Before_rad_clinic_signature	Points	Before_dl_signature	Points
0	0	0	0
0.2	20	1	38
0.3	30	gender	Points
0.5	50	women	0
0.6	60	men	19
0.7	70	N stage	Points
0.8	80	0	8
0.9	90	1	4
1	100	2	0

**Table 18:** GPR probability corresponding to different total scores in the before_nomogram.

Total Points	Probability of GPR
79	0.1
84	0.3
92	0.7
97	0.9

#### Drawing and calibration of the after_nomogram

**Figure 15:**
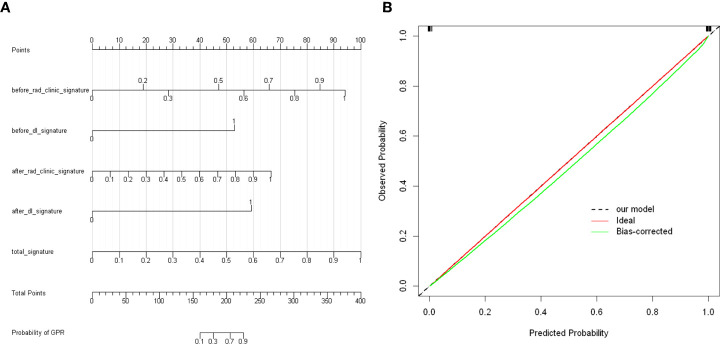
**(A)** After_nomogram.Locate on the before_rad_clinic_signature, before_dl_signature, after_rad_clinic_signature, after_dl_signature, and entire_signature coordinate axis. Draw a straight line perpendicular to the first points, calculate and sum the scores corresponding to each straight line, locate on the total points coordinate axis, and draw a straight line perpendicular to the horizontal axis of the probability of GPR. The corresponding value is the probability of pathological response to GPR in patients with non-small cell lung cancer after neoadjuvant immunotherapy. **(B)** Calibration curve corresponding to after_nomogram. The consistency between the probability of GPR predicted by the nomogram after 1,000 repeated samplings and the actual GPR. The 45° red line represents the ideal prediction performance. The black dotted line and the solid green line represent the prediction performance of the nomogram and the correction of the deviation of the nomogram, respectively. The closer the black dotted line is to the 45° ideal red line, the higher the model’s prediction accuracy.

In our study, we used previously constructed before_rad_clinic_signature, before_dl_signature, after_rad_clinic_signature, after_dl_signature, and entire_signature to jointly construct a posttreatment nomogram (after_nomogram). According to the characteristics in **Figure 15A**, a patient’s corresponding probability of GPR after treatment can be calculated. **Table 19** shows the features in the after_nomogram corresponding scores. **Table 20** shows the GPR probability to different total scores in the after_nomogram. **Figure 15B** shows that the probability of GPR predicted by the nomogram after 1,000 repeated samplings and the actual GPR is consistent.

**Table 19:** The features in the nomogram and the corresponding scores.

Before_rad_clinic_signature	Points	After_rad_clinic_signature	Points
0	0	0	0
0.2	19	0.1	7
0.3	28	0.2	13
0.5	47	0.3	20
0.6	56	0.4	27
0.7	66	0.5	33
0.8	75	0.6	40
0.9	85	0.7	47
1	94	0.8	53
		0.9	60
		1	67
after_dl_signature	Points	total_signature	Points
0	0	0	0
1	59	0.1	10
before_dl_signature	Points	0.2	20
0	0	0.3	30
1	53	0.4	40
		0.5	50
		0.6	60
		0.7	70
		0.8	80
		0.9	90
		1	100

**Table 20:** GPR probability corresponding to different total scores in the after_nomogram.

Total Points	Probability of GPR
160	0.1
180	0.3
205	0.7
225	0.9

## Discussion

Lung cancer is still the leading cancer in the world and in China, where NSCLC is the most common, with an incidence rate of about 85% (2, 3). Surgery is still the main treatment for early and locally advanced NSCLC (I, II and IIIA, IIIB)(NCCN) (4, 5). However, great progress has been made in recent years with the application of ICIs as NAT approach. Immunotherapy-based NAT (nivolumab 360 mg and platinum-containing chemotherapy, once every 3 weeks, for three cycles) has been included in the latest NCCN guidelines(NCCN) (4, 5). Although PD-L1 and tumor mutation burden (TMB) can indicate the effect of immunotherapy to some extent (32), different types of immune cells in the tumor microenvironment, such as CD8+ T-cell infiltration, usually indicate that immunotherapy confers a good response and prognosis (33).

The combination of different immune cells, such as CD3/CD8/CD45RO combined with immune score (34), has a certain suggestive effect on the efficacy of immunotherapy. However, there is currently no reliable indicator to predict the exact efficacy of immunotherapy-based NAT. The common endpoints of clinical trials are progression-free survival (PFS) and overall survival (OS). Although the pathological response can evaluate the treatment benefit earlier than the traditional clinical trial endpoints, the histopathological evaluation can only be determined after the pathological results of surgical resection. CT chest is a common diagnostic imaging modality of lung cancer.

In contrast, another common efficacy evaluation standard is RECIST 1.1, which is mainly based on the 2D evaluation of the number and size of tumors shown on CT (35). From a quantitative point of view, this method is basic and ignores a large amount of information in medical images. Based on saving diagnostic financial costs, radiomics uses high-throughput technology to extract the conventional features and/or DL features of medical images together with molecular biological information such as genes, proteins, and tumor metabolism, which is then transformed into quantitative features. Combined with machine learning or/and DL, it can be used for disease diagnosis, therapeutic efficacy prediction, and prognosis analysis (22, 23, 25, 26).

Accurate prediction of treatment response is of great significance for the stratification and selection of patients benefiting from immunotherapy. Yang et al. (36) selected 88 radiomics features from the CT images of 92 patients with lung cancer before immunotherapy and constructed a random forest model. Combined with clinicopathological information, they successfully predicted the patients who would benefit from ICI treatment (the AUCs of the training and validation groups were 0.848 and 0.795, respectively). Similarly, Barabino et al. (37) extracted the radiomics features of lung lesions from CT scans at baseline and the first evaluation and calculated their changes by absolute difference and relative reduction (Delta, Δ). After feature screening and model construction, 27 delta features were identified, which were able to distinguish the response to NSCLC immunotherapy with statistically significant accuracy. Moreover, it was found that the changes in the other nine features were significantly correlated with false progression. Another report by Shen et al. (38) predicted the effect of immunotherapy in NSCLC patients through texture feature extraction and texture analysis of lung enhanced CT before treatment. The highest prediction efficiency was 88.2% (sensitivity), 76.3% (specificity), and 81.9% accuracy. These studies suggest that radiomics can help predict and select the right NSCLC patient population for immunotherapy. Before immunotherapy was widely used in the clinic, there have been studies using radiomics to predict the pathological response after concurrent neoadjuvant chemoradiotherapy. A study by Coroller et al. (39) showed that both radiomics features of primary tumors and lymph node phenotypic information could predict pathological responses. Also, in another study (40), they found that seven features could predict pathological gross residual lesions (AUC >0.6, p < 0.05), in which one indicator could predict pathological complete response (AUC = 0.63, p = 0.01), and tumors with poor response to neoadjuvant chemoradiotherapy were more likely to show well-circumscribed (spherical nonproportional, AUC = 0.63, p = 0.009) and spiculated lesions (LoG 5 mm 3D-GLCM entropy, AUC = 0.61, p = 0.03). At present, there is no relevant research on radiomics that predicts the efficacy of neoadjuvant immunotherapy. To explore the role of radiomics in predicting the pathological remission of NSCLC after neoadjuvant immunotherapy, this study extracted the conventional radiological features and DL features from the CT images of NSCLC patients before and after neoadjuvant immunotherapy combined with clinicopathological information to construct models that can predict the pathological remission of NSCLC patients after immunotherapy-based NAT.

The before_rad_cil model was constructed after feature screening. Four radiomics traditional features (“original_shape_Flatness, ” “wavelet-HHL_firstorder_Skewness, ” “wavelet-HLH_firstorder_Skewness, ” and “wavelet-LLL_glcm_Correlation”) and two clinical features (“sex” and “N stage”) were obtained. Eight common machine learning algorithms model the selected features: SVM, KNN, DecisionTree, RandomForest, ExtraTrees, XGBoost, LightGBM, and MLP. After 100 random groupings of 5-fold cross-validation, the average prediction accuracy of the KNN model was the highest, 0.708. Conventional radiomics features were extracted from the CT images of NSCLC patients after immunotherapy-based NAT and combined with the clinicopathological information obtained after immunotherapy-based NAT. The after_rad_cil model was constructed. After feature screening, seven radiomics traditional features (“exponential_firstorder_Skewness,” “exponential_glrlm_RunEntropy,” “log-sigma-5-0-mm-3D_firstorder_Kurtosis,” “logarithm_firstorder_Skewness,” “original_shape_Elongation,” “original_shape_Flatness,” and “wavelet-LLH_glcm_ClusterShade”) and two clinical features (“after_CRP” and “after_percentage of lymphocytes”) were obtained.

After the selected features were modeled and cross-verified by eight common machine learning algorithms, the average prediction accuracy of the SVM model was the highest (0.682). After fine-tuning resnet50, the before_dl model extracted DL features from the CT images of NSCLC patients before immunotherapy-based NAT. The DL features only reflected the relationship between features and outcomes without exact physical meaning. Eight common machine learning algorithms then modeled the features after dimensionality reduction by PCA: SVM, KNN, DecisionTree, RandomForest, ExtraTrees, XGBoost, LightGBM, and MLP. After 100 random groupings of 5-fold cross-validation, the average prediction accuracy of the SVM model was the highest, 0.629.

In the after_dl model constructed by the DL features extracted from the CT images of patients after immunotherapy-based NAT like the before_dl model, the average prediction accuracy of SVM was the highest (0.603). The entire model was a prediction model constructed by combining the conventional features of radiomics, DL features, and clinicopathological features before and after NAT. After being modeled by eight common machine learning algorithms and 100 random groupings of 5-fold cross-validation, the average prediction accuracy of the MLP model was the highest, which was 0.805.

Nomograms can graphically describe biological information, characteristics, and clinical variables as a statistical prediction model and estimate the individualized risk according to the characteristics of patients and diseases. It is a simple, easy-to-understand, and user-friendly clinical decision-making tool (41) and was widely used in individualized prognostic evaluation of breast cancer (42), rectal cancer (43), prostate cancer (44), glioma (45), and lung adenocarcinoma (46).

We combined prognostic variables obtained in the before_rad_clinic model, gender, N stage, before_ rad_ clinic_ signature, and before_dl_signature, to construct the before_nomogram, while the before_rad_clinic_signature, before_dl_signature, after_rad_clinic_signature, after_dl_signature, and entire_signature were used to construct the after_nomogram. The calibration curve showed that the nomogram before and after treatment had a good predictive effect on the GPR.

Our results suggest that radiomics can predict the pathological remission of NSCLC after immunotherapy-based NAT. Similar to previous studies (19, 47–49), the prediction efficiency of the entire model is higher than that of the single DL model and the radiomics traditional features combined clinical features model. CT images and clinicopathological information obtained before NAT were constructed as the before_rad_cil model. After classification using the KNN algorithm, the average prediction accuracy was 0.708. Combining GPR-related clinical variables was done to construct the before_nomogram. It shows that clinicians can judge the probability of achieving GPR before treatment in each patient who intends to receive immunotherapy-based NAT.

The entire model had the highest predictive efficacy after classification using the MLP algorithm with an average predictive accuracy of 0.805, combined with the before_rad_clinic_signature, before_dl_signature, after_rad_clinic_signature, and after_dl_signature to construct the after_nomogram, which can predict the probability that patients are obtaining GPR after immunotherapy-based NAT ahead of surgery. If the follow-up data show that patients who achieved GPR after immunotherapy-based NAT have significant survival benefits after surgery or not, reducing the scope of surgery or avoiding surgical treatment altogether may have the same survival benefits as patients with total resection or extended resection.

Although the results are satisfactory, our study also has some limitations. First, the sample size of this retrospective study is limited, and the consistency test between observers was not carried out. Although we used various methods for feature selection and compared the prediction results of various machine learning methods to select the best model, the limited data set may lead to insufficient generalization of the model. Therefore, in future research, we will strive to conduct a multicenter study and aim to construct large samples with diversified data sets to evaluate the proposed model and verify the robustness and effectiveness of our model through prospective studies.

Secondly, previous studies (50) have shown that adenocarcinoma (ADC) and squamous cell carcinoma (SCC) have different imaging phenotypes on CT scans: peripheral hair glass shadows are more common in ADC, and SCC is more likely to show necrosis. Different imaging phenotypes may lead to different prediction performances. Due to the limited number of cases in this study, the data sets of different histopathological types (ADC and SCC) cannot be hierarchically modeled and verified.

The model constructed by merging the two tumors may lead to a decline in prediction efficiency. Therefore, larger data sets should be used in future research, and the two histological subtypes should be hierarchically modeled and verified. This study only analyzed the predictive effect of some clinicopathological features, conventional radiomics features, and DL features extracted from CT images before and after immunotherapy-based NAT for NSCLC. Studies have shown that the pathological characteristics (51) of patients, genes (52), and protein expression (53) can also affect the prognosis. In future studies, if we combine pathology, genomics, proteomics, and comprehensive clinical information, it is expected to further improve the prediction efficiency of the model. Finally, another limitation of this study is manually sketching the ROI, in which the operator may have different sketching regions, which is time-consuming and laborious. Some studies (54, 55) have begun to attempt to automatically sketch ROIs and automatically extract conventional radiomics features and DL features to construct end-to-end models (56) to complete the research objectives. In case the constructed model has stable performance and accurate efficacy, it may give patients a relatively accurate prediction within a few minutes after obtaining patient-related information, which is convenient for clinical application and makes precise individualized treatment possible.

## Conclusion

CT-based radiomics has a good predictive ability for GPR in NSCLC patients receiving immunotherapy-based NAT. Among the radiomics features combined with the clinicopathological information model, DL feature model, and the entire model, the entire model had the highest prediction accuracy.

## Data availability statement

The original contributions presented in the study are included in the article/**Supplementary Material**. Further inquiries can be directed to the corresponding author.

## Ethics statement

Ethical review and approval was not required for the study on human participants in accordance with the local legislation and institutional requirements. Written informed consent for participation was not required for this study in accordance with the national legislation and the institutional requirements.

## Author contributions

LQ, YK and HJ contributed to conception and design of the study. QS and YK organized the database. LQ performed the statistical analysis. LQ wrote the draft of the manuscript. All authors contributed to the article and approved the submitted version.

## Funding

This work was supported by the Natural Science Foundation of Hunan Province, China (Grand NO.2020JJ4915) and the Postgraduate Independent Exploration and Innovation Project of Central South University (No. 2022ZZTS0905).

## Acknowledgments

For editing the language of this manuscript, we thank Omar, Ph.D., and for advice regarding the code used in this manuscript, we thank the OnekeyAl platform and its developers.

## Conflict of interest

The authors declare that the research was conducted in the absence of any commercial or financial relationships that could be construed as a potential conflict of interest.

The handling editor PY declared a shared parent affiliation with the authors at the time of review.

## Publisher’s note

All claims expressed in this article are solely those of the authors and do not necessarily represent those of their affiliated organizations, or those of the publisher, the editors and the reviewers. Any product that may be evaluated in this article, or claim that may be made by its manufacturer, is not guaranteed or endorsed by the publisher.

## References

[1] Yang D Liu Y Bai C Wang X Powell CA. Epidemiology of lung cancer and lung cancer screening programs in China and the united states. Cancer Lett (2020) 468:82–7. doi: 10.1016/j.canlet.2019.10.009 31600530

[2] Chen W Zheng R Baade PD Zhang S Zeng H Bray F. Cancer statistics in China 2015. CA Cancer J Clin (2016) 66(2):115–32. doi: 10.3322/caac.21338 26808342

[3] Sung H Ferlay J Siegel RL Laversanne M Soerjomataram I Jemal A. Global cancer statistics 2020: GLOBOCAN estimates of incidence and mortality worldwide for 36 cancers in 185 countries. CA Cancer J Clin (2021) 71(3):209–49. doi: 10.3322/caac.21660 33538338

[4] NCCN. NCCN clinical practice guidelines in oncology non-small cell lung Cancer(Version 2.2022–march 7,2022). USA: NCCN (2022). Available at: http://www.nccn.org/. 10.6004/jnccn.2021.0058 PMC10203822 34902832

[5] NCCN. "NCCN clinical practice guidelines in oncology non-small cell lung Cancer(Version 3.2022–march 16,2022)". USA: NCCN (2022). Available at: http://www.nccn.org/. 10.6004/jnccn.2022.0025 35545176

[6] Bunn P Schenk E Pacheco J Dimou AJO. New developments in neoadjuvant therapy for lung cancer. Oncology (2019) 33(3):101–106, 109. 30866032

[7] Qu Y Emoto K Eguchi T Aly RG Zheng H Chaft JE. Pathologic assessment after neoadjuvant chemotherapy for NSCLC: Importance and implications of distinguishing adenocarcinoma from squamous cell carcinoma. J Thorac Oncol (2019) 14(3):482–93. doi: 10.1016/j.jtho.2018.11.017 PMC6382593 30503889

[8] de Silva M Itchins M Pavlakis N. Breakthrough 5-year survival with pembrolizumab in keynote-001 study: Horizon shifting in advanced non-small cell lung cancer with immune check point inhibition. Ann Transl Med (2020) 8(8):555. doi: 10.21037/atm.2020.01.87 32411778 PMC7214900

[9] Socinski MA Jotte RM Cappuzzo F Orlandi F Stroyakovskiy D Nogami N. Atezolizumab for first-line treatment of metastatic nonsquamous NSCLC. N Engl J Med (2018) 378(24):2288–301. doi: 10.1056/NEJMoa1716948 29863955

[10] Ikeda S Kato T Kenmotsu H Ogura T Iwasawa S Iwasawa T. A phase II study of atezolizumab for pretreated advanced/recurrent non-small cell lung cancer with idiopathic interstitial pneumonias: rationale and design for the TORG1936/AMBITIOUS study. Ther Adv Med Oncol (2020) 12:1758835920922022. doi: 10.1177/1758835920922022 32426051 PMC7222231

[11] Forde PM Chaft JE Smith KN Anagnostou V Cottrell TR Hellmann MD. Neoadjuvant PD-1 blockade in resectable lung cancer. N Engl J Med (2018) 378(21):1976–86. doi: 10.1056/NEJMoa1716078 PMC6223617 29658848

[12] Cascone T William WN Weissferdt A Lin HY Sepesi B. Neoadjuvant nivolumab (N) or nivolumab plus ipilimumab (NI) for resectable non-small cell lung cancer (NSCLC): Clinical and correlative results from the NEOSTAR study. J Clin Oncol (2019) 37(Suppl 15):8504–4. doi: 10.1200/JCO.2019.37.15_suppl.8504

[13] Provencio M Nadal E Insa A García-Campelo M. Neoadjuvant chemotherapy and nivolumab in resectable non-small-cell lung cancer (NADIM): an open-label, multicentre, single-arm, phase 2 trial. Oncology (2020) 21(11):1413–22. doi: 10.1016/S1470-2045(20)30453-8 32979984

[14] Zhi Z.B.L.X.Z. Expert consensus on the pathological evaluation of neoadjuvant therapy efficacy for non-small cell lung cancer. Chin J Pathol (2021) 50(9):1002–7. doi: 10.3760/cma.j.cn112151-20210429-00335 34496489

[15] Hellmann MD Chaft JE William WN Jr. Rusch V Pisters KM Kalhor N. Pathological response after neoadjuvant chemotherapy in resectable non-small-cell lung cancers: proposal for the use of major pathological response as a surrogate endpoint. Lancet Oncol (2014) 15(1):e42–50. doi: 10.1016/s1470-2045(13)70334-6 PMC4734624 24384493

[16] Travis WD Dacic S Wistuba I Sholl L Adusumilli P Bubendorf L. IASLC multidisciplinary recommendations for pathologic assessment of lung cancer resection specimens after neoadjuvant therapy. J Thorac Oncol (2020) 15(5):709–40. doi: 10.1016/j.jtho.2020.01.005 PMC8173999 32004713

[17] Lambin P Rios-Velazquez E Leijenaar R Carvalho S van Stiphout RG Granton P. Radiomics: extracting more information from medical images using advanced feature analysis. Eur J Cancer (2012) 48(4):441–6. doi: 10.1016/j.ejca.2011.11.036 PMC4533986 22257792

[18] Guo J Liu Z Shen C Li Z Yan F Tian J. MR-based radiomics signature in differentiating ocular adnexal lymphoma from idiopathic orbital inflammation. Eur Radiol (2018) 28(9):3872–81. doi: 10.1007/s00330-018-5381-7 29632999

[19] Lv J Chen X Liu X Du D Lv W Lu L. Imbalanced data correction based PET/CT radiomics model for predicting lymph node metastasis in clinical stage T1 lung adenocarcinoma. Front Oncol (2022) 12:788968. doi: 10.3389/fonc.2022.788968 35155231 PMC8831550

[20] Rossi G Barabino E Fedeli A Ficarra G Coco S Russo A. Radiomic detection of EGFR mutations in NSCLC. Cancer Res (2021) 81(3):724–31. doi: 10.1158/0008-5472.Can-20-0999 33148663

[21] Song L Zhu Z Mao L Li X Han W Du H. Clinical, conventional CT and radiomic feature-based machine learning models for predicting ALK rearrangement status in lung adenocarcinoma patients. Front Oncol (2020) 10:369. doi: 10.3389/fonc.2020.00369 32266148 PMC7099003

[22] Hirose TA Arimura H Ninomiya K Yoshitake T Fukunaga JI Shioyama Y. Radiomic prediction of radiation pneumonitis on pretreatment planning computed tomography images prior to lung cancer stereotactic body radiation therapy. Sci Rep (2020) 10(1):20424. doi: 10.1038/s41598-020-77552-7 33235324 PMC7686358

[23] Bortolotto C Lancia A Stelitano C Montesano M Merizzoli E Agustoni F. Radiomics features as predictive and prognostic biomarkers in NSCLC. Expert Rev Anticancer Ther (2021) 21(3):257–66. doi: 10.1080/14737140.2021.1852935 33216651

[24] Wu Y Li T Han Y Jiang J. Use of radiomic features and support vector machine to discriminate subjective cognitive decline and healthy controls(). Annu Int Conf IEEE Eng Med Biol Soc (2020) 2020:1762–5. doi: 10.1109/embc44109.2020.9175840 33018339

[25] Wang D Huang C Bao S Fan T Sun Z Wang Y. Study on the prognosis predictive model of COVID-19 patients based on CT radiomics. Sci Rep (2021) 11(1):11591. doi: 10.1038/s41598-021-90991-0 34078950 PMC8172890

[26] Wang H Wang L Lee EH Zheng J Zhang W Halabi S. Decoding COVID-19 pneumonia: comparison of deep learning and radiomics CT image signatures. Eur J Nucl Med Mol Imaging (2021) 48(5):1478–86. doi: 10.1007/s00259-020-05075-4 PMC7581467 33094432

[27] Wang S Shi J Ye Z Dong D Yu D Zhou M. Predicting EGFR mutation status in lung adenocarcinoma on computed tomography image using deep learning. Eur Respir J (2019) 53(3):1800986. doi: 10.1183/13993003.00986-2018 30635290 PMC6437603

[28] Choi YS Bae S Chang JH Kang SG Kim SH Kim J. Fully automated hybrid approach to predict the IDH mutation status of gliomas *via* deep learning and radiomics. Neuro Oncol (2021) 23(2):304–13. doi: 10.1093/neuonc/noaa177 PMC7906063 32706862

[29] Uhm KH Jung SW Choi MH Shin HK Yoo JI Oh SW. Deep learning for end-to-end kidney cancer diagnosis on multi-phase abdominal computed tomography. NPJ Precis Oncol (2021) 5(1):54. doi: 10.1038/s41698-021-00195-y 34145374 PMC8213852

[30] Saba L Biswas M Kuppili V Cuadrado Godia E Suri HS Edla DR. The present and future of deep learning in radiology. Eur J Radiol (2019) 114:14–24. doi: 10.1016/j.ejrad.2019.02.038 31005165

[31] Bo L Zhang Z Jiang Z Yang C Huang P Chen T. Differentiation of brain abscess from cystic glioma using conventional MRI based on deep transfer learning features and hand-crafted radiomics features. Front Med (Lausanne) (2021) 8:748144. doi: 10.3389/fmed.2021.748144 34869438 PMC8636043

[32] Kaderbhaï C Tharin Z Ghiringhelli F. The role of molecular profiling to predict the response to immune checkpoint inhibitors in lung cancer. Cancers (Basel) (2019) 11(2):201. doi: 10.3390/cancers11020201 PMC6406957 30744168

[33] Jiang X Xu J Liu M Xing H Wang Z Huang L. Adoptive CD8(+) T cell therapy against cancer:Challenges and opportunities. Cancer Lett (2019) 462:23–32. doi: 10.1016/j.canlet.2019.07.017 31356845

[34] Nosho K Baba Y Tanaka N Shima K Hayashi M Meyerhardt JA. Tumour-infiltrating T-cell subsets, molecular changes in colorectal cancer, and prognosis: cohort study and literature review. J Pathol (2010) 222(4):350–66. doi: 10.1002/path.2774 PMC3033700 20927778

[35] Eisenhauer EA Therasse P Bogaerts J Schwartz LH Sargent D Ford R. New response evaluation criteria in solid tumours: revised RECIST guideline (version 1.1). Eur J Cancer (2009) 45(2):228–47. doi: 10.1016/j.ejca.2008.10.026 19097774

[36] Yang B Zhou L Zhong J Lv T Li A Ma L. Combination of computed tomography imaging-based radiomics and clinicopathological characteristics for predicting the clinical benefits of immune checkpoint inhibitors in lung cancer. Respir Res (2021) 22(1):189. doi: 10.1186/s12931-021-01780-2 34183009 PMC8240400

[37] Barabino E Rossi G Pamparino S Fiannacca M Caprioli S Fedeli A. Exploring response to immunotherapy in non-small cell lung cancer using delta-radiomics. Cancers (Basel) (2022) 14(2):350. doi: 10.3390/cancers14020350 35053513 PMC8773717

[38] Shen L Fu H Tao G Liu X Yuan Z Ye X. Pre-immunotherapy contrast-enhanced CT texture-based classification: A useful approach to non-small cell lung cancer immunotherapy efficacy prediction. Front Oncol (2021) 11:591106. doi: 10.3389/fonc.2021.591106 33968716 PMC8103028

[39] Coroller TP Agrawal V Huynh E Narayan V Lee SW Mak RH. Radiomic-based pathological response prediction from primary tumors and lymph nodes in NSCLC. J Thorac Oncol (2017) 12(3):467–76. doi: 10.1016/j.jtho.2016.11.2226 PMC5318226 27903462

[40] Coroller TP Agrawal V Narayan V Hou Y Grossmann P Lee SW. Radiomic phenotype features predict pathological response in non-small cell lung cancer. Radiother Oncol (2016) 119(3):480–6. doi: 10.1016/j.radonc.2016.04.004 PMC4930885 27085484

[41] Balachandran VP Gonen M Smith JJ DeMatteo RP. Nomograms in oncology: more than meets the eye. Lancet Oncol (2015) 16(4):e173–180. doi: 10.1016/s1470-2045(14)71116-7 PMC4465353 25846097

[42] Li F Zhao Y Wei Y Xi Y Bu H. Tumor-infiltrating lymphocytes improve magee equation-based prediction of pathologic complete response in HR-Positive/HER2-Negative breast cancer. Am J Clin Pathol (2022) 158(2):291–9. doi: 10.1093/ajcp/aqac041 35486808

[43] Jiang W Wang S Wan J Zheng J Dong X Liu Z. Association of the collagen signature with pathological complete response in rectal cancer patients. Cancer Sci (2022) 113(7):2409–24. doi: 10.1111/cas.15385 PMC9277261 35485874

[44] Rocco B Sighinolfi MC Sandri M Puliatti S Bianchi G. A novel nomogram for predicting ECE of prostate cancer. BJU Int (2018) 122(6):916–8. doi: 10.1111/bju.14503 30460784

[45] Li J Wang J Ding Y Zhao J Wang W. Prognostic biomarker SGSM1 and its correlation with immune infiltration in gliomas. BMC Cancer (2022) 22(1):466. doi: 10.1186/s12885-022-09548-7 35484511 PMC9047296

[46] Zhou M Zhu X. Construction and validation of a robust ferroptosis-associated gene signature predictive of prognosis in lung adenocarcinoma. Med (Baltimore) (2022) 101(16):e29068. doi: 10.1097/md.0000000000029068 PMC9276120 35482981

[47] Cong M Feng H Ren JL Xu Q Cong L Hou Z. Developing a predictive radiomics model for lymph node metastases in pre-surgical CT-based stage IA non-small cell lung cancer. Lung Cancer (2020) 139:73–9. doi: 10.1016/j.lungcan.2019.11.003 31743889

[48] Hu X Gong J Zhou W Li H Wang S Wei M. Computer-aided diagnosis of ground glass pulmonary nodule by fusing deep learning and radiomics features. Phys Med Biol (2021) 66(6):065015. doi: 10.1088/1361-6560/abe735 33596552

[49] Ren C Zhang J Qi M Zhang J Zhang Y Song S. Machine learning based on clinico-biological features integrated (18)F-FDG PET/CT radiomics for distinguishing squamous cell carcinoma from adenocarcinoma of lung. Eur J Nucl Med Mol Imaging (2021) 48(5):1538–49. doi: 10.1007/s00259-020-05065-6 PMC8113203 33057772

[50] Liu J Cui J Liu F Yuan Y Guo F Zhang G. Multi-subtype classification model for non-small cell lung cancer based on radiomics: SLS model. Med Phys (2019) 46(7):3091–100. doi: 10.1002/mp.13551 31002395

[51] Bera K Schalper KA Rimm DL Velcheti V Madabhushi A. Artificial intelligence in digital pathology - new tools for diagnosis and precision oncology. Nat Rev Clin Oncol (2019) 16(11):703–15. doi: 10.1038/s41571-019-0252-y PMC6880861 31399699

[52] McDonald BR Contente-Cuomo T Sammut SJ Odenheimer-Bergman A Ernst B Perdigones N. Personalized circulating tumor DNA analysis to detect residual disease after neoadjuvant therapy in breast cancer. Sci Transl Med (2019) 11(504):eaax7392. doi: 10.1126/scitranslmed.aax7392 31391323 PMC7236617

[53] Huang CM Huang CW Ma CJ Yeh YS Su WC Chang TK. Predictive value of FOLFOX-based regimen, long interval, hemoglobin levels and clinical negative nodal status, and postchemoradiotherapy CEA levels for pathological complete response in patients with locally advanced rectal cancer after neoadjuvant chemoradiotherapy. J Oncol (2020) 2020:9437684. doi: 10.1155/2020/9437684 32411245 PMC7204332

[54] Heutink F Koch V Verbist B van der Woude WJ Mylanus E Huinck W. Multi-scale deep learning framework for cochlea localization, segmentation and analysis on clinical ultra-high-resolution CT images. Comput Methods Programs BioMed (2020) 191:105387. doi: 10.1016/j.cmpb.2020.105387 32109685

[55] Zhou X. Automatic segmentation of multiple organs on 3D CT images by using deep learning approaches. Adv Exp Med Biol (2020) 1213:135–47. doi: 10.1007/978-3-030-33128-3_9 32030668

[56] Jin C Yu H Ke J Ding P Yi Y Jiang X. Predicting treatment response from longitudinal images using multi-task deep learning. Nat Commun (2021) 12(1):1851. doi: 10.1038/s41467-021-22188-y 33767170 PMC7994301

